# Multi-omics analysis reveal clinical-gut-brain interactions in female ibs patients with adverse childhood experiences

**DOI:** 10.1186/s13293-025-00757-w

**Published:** 2025-11-25

**Authors:** Michelle Binod, Lin Chang, Ming Wei Hung, Tien S. Dong, Lisa A. Kilpatrick, Anthony Tomasevic, Michelle Choy, Andrea Shin, Emeran A. Mayer, Arpana Church

**Affiliations:** 1 Division of Pediatric Gastroenterology, Hepatology and Nutrition, UCLA, Los Angeles, CA USA; 2 G. Oppenheimer Center for Neurobiology of Stress and Resilience, UCLA, Los Angeles, CA USA; 3 Goodman-Luskin Microbiome Center, UCLA, Los Angeles, CA USA; 4 Vatche and Tamar Manoukian Division of Digestive Diseases, UCLA, Los Angeles, CA USA; 5 David Geffen School of Medicine, UCLA, Los Angeles, CA USA; 6 University of California, Los Angeles, CA USA

**Keywords:** Adverse childhood experiences, Irritable bowel syndrome, Brain-gut-microbiome system, Multi-omics, Sex differences, Stress, Salience network, *Akkermansia*, *Bifidobacterium*

## Abstract

**Background:**

The brain-gut system, which involves bidirectional communication between the central nervous system and the gut, plays a central role in stress responses. Its dysregulation is implicated in irritable bowel syndrome (IBS), a stress-sensitive, female-predominant disorder characterized by abdominal pain and altered bowel habits. Adverse childhood experiences (ACE) increase the risk and severity of IBS, likely by amplifying stress responsiveness and gut-brain dysfunction in females. However, the mechanisms involved are unknown.

**Aim:**

This study aimed to identify a multi-omic signature linking ACE exposure to IBS females via clinical, neuroimaging, and gut microbiome features as compared to healthy control (HC) females.

**Methods:**

Data was analyzed from participants with Rome positive IBS and HCs. Four subgroups were created based on IBS diagnosis and ACE score with high ACE defined as ≥2 and low as ACE 0-1. Validated questionnaires assessed clinical variables. Biological markers included multimodal brain MRI, and gut microbial function using metagenomics. eXtreme gradient boosting (XGBoost) identified key differentiating features between the groups. Connectograms visualized relationships across mutli-omics data within each group.

**Results:**

Among 188 female participants, the four groups included IBS with high ACE (n=37), IBS with low ACE (n=55), HCs with high ACE (n=19), and HCs with low ACE (n=77). Key findings include: 1. High ACE participants with IBS versus their HC counterparts showed increased depression and anxiety symptoms, GI-symptom related anxiety, perceived stress, somatic symptom severity, and poorer physical and mental health scores. 2. High ACE participants with IBS had negative associations between key bacteria such as *Akkermansia* (a beneficial bacteria) and somatic symptom severity, and between *Bifidobacterium* and ACE parental divorce/separation and alterations in the salience and central autonomic networks. 3. The ensemble model accurately distinguished IBS patients with high ACE (AUC of 0.87), demonstrating strong predictive performance with an overall model accuracy of 78%.

**Conclusions:**

Our findings highlight the unique microbiota and brain networks contributing to a complex interplay of chronic stress as measured by early life adversity, the brain-gut-microbiome system, and IBS pathophysiology which can inform therapeutic targets aimed at mitigating the long-term impacts of early life stress in female IBS patients.

**Graphical abstract:**

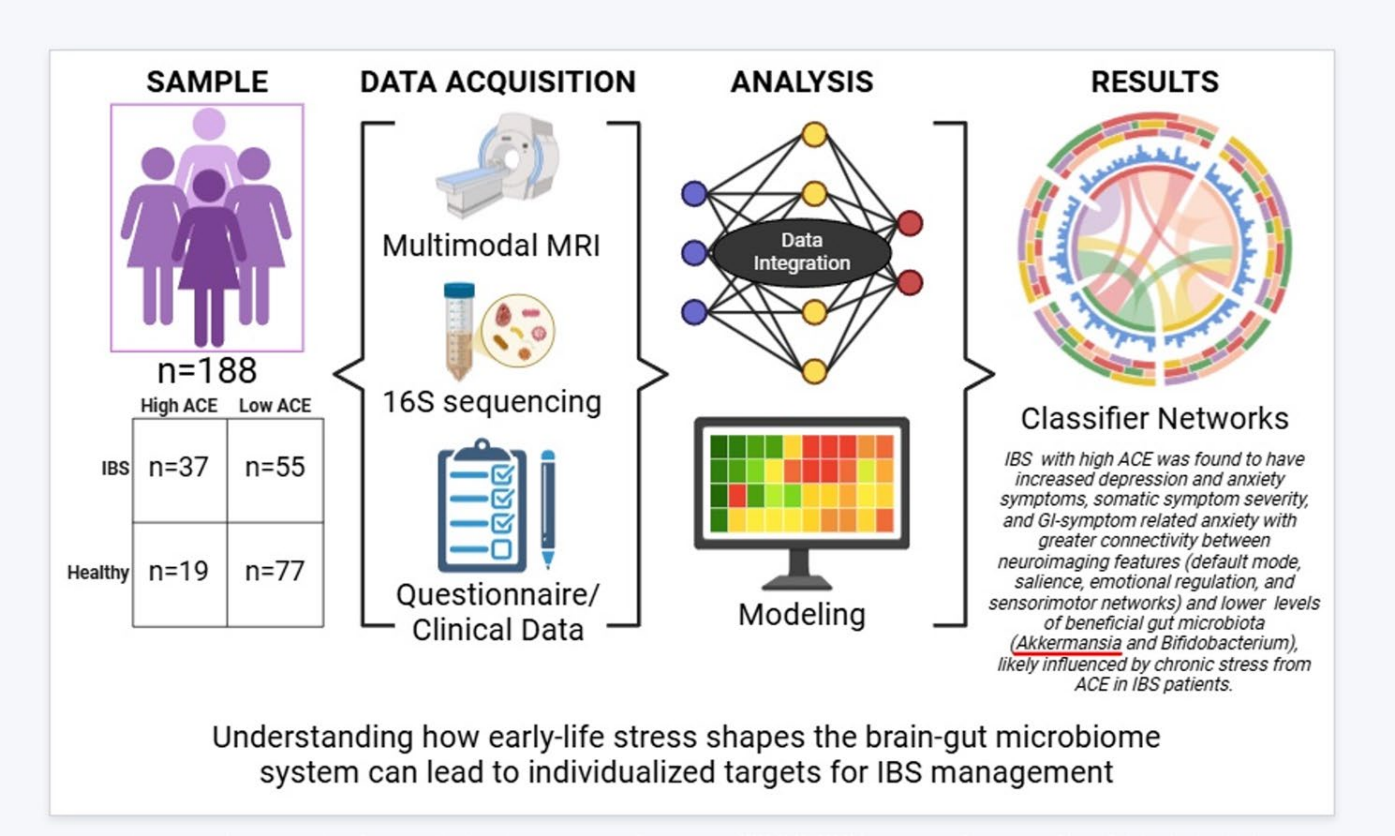

## Abstract (plain language summary)

Irritable Bowel Syndrome (IBS) is a common, female-predominant disorder of gut brain interactions, often linked to stress and early life adversity. This study explores how adverse childhood experiences (ACE) impact IBS symptoms, brain structure and function, and gut microbiome composition in women. We studied 188 premenopausal women, dividing them into groups based on IBS diagnosis and early life adversity history. Using a machine-learning approach, we identified key biological signatures associated with IBS and ACE. Our findings show that women with IBS and high ACE scores have unique brain connectivity patterns, altered gut bacteria, and greater somatic symptom severity compared to those with low ACE scores or healthy controls. Specifically, differences in brain regions related to stress and pain processing, along with changes in gut bacteria linked to inflammation and digestion, highlight the complex interactions within the brain-gut-microbiome system. These results reinforce the concept that IBS is a complex disorder influenced by multiple biological systems. This study highlights the importance of understanding how early-life stress shapes the brain-gut microbiome system and identifies potential targets for improving IBS management and overall patient well-being.

Adverse childhood experiences are strongly linked to IBS, affect brain structure and function, gut microbiome, and symptom severity.

IBS is also more common in women, potentially due to the influence of adverse childhood experiences on the brain-gut microbiome system.

Our study analyzed 188 premenopausal women with IBS or healthy controls using clinical assessments, gut microbiome sequencing, and brain imaging.

Machine-learning models identified unique IBS brain-gut signatures, highlighting increased somatic symptom severity, gut bacterial changes, and brain connectivity alterations in women with IBS and high ACE.

Key microbiome changes included reduced beneficial bacteria.

Neuroimaging findings showed alterations in stress and pain processing brain regions, particularly in women with high ACE.

## Highlights

Adverse childhood experiences are strongly linked to IBS, affect brain structure and function, gut microbiome, and symptom severity.

IBS is also more common in women, potentially due to the influence of adverse childhood experiences on the brain-gut microbiome system.

Our study analyzed 188 premenopausal women with IBS or healthy controls using clinical assessments, gut microbiome sequencing, and brain imaging.

Machine-learning models identified unique IBS brain-gut signatures, highlighting increased somatic symptom severity, gut bacterial changes, and brain connectivity alterations in women with IBS and high ACE.

Key microbiome changes included reduced beneficial bacteria.

Neuroimaging findings showed alterations in stress and pain processing brain regions, particularly in women with high ACE.

## Introduction

Irritable Bowel Syndrome (IBS) is a stress-sensitive, female-predominant disorder characterized by chronic abdominal pain and altered bowel habits. Evidence supports that IBS is a disorder of gut-brain Interactions (DGBI), which can be attributed to dysregulation of central nervous system (CNS) processing, dysbiosis of the gut microbiota, altered mucosal and immune function, motility disturbance, and visceral hypersensitivity [1]. There is a higher prevalence of IBS in women compared to men across various diagnostic criteria with an odds ratio of 1.67 (95% CI: 1.53–1.82.53.82) [2]. Estrogen and progesterone, the primary female sex hormones, have been shown to modulate gastrointestinal (GI) function and visceral sensitivity, potentially worsening IBS symptoms [3, 4]. These hormones impact the brain-gut system, influencing gut motility, pain perception, and stress responses, all of which are central to IBS pathophysiology [4].

Adverse childhood experiences (ACEs) are strongly associated with the development and exacerbation of IBS symptoms, mediated through psychological factors such as anxiety, depression, and stress response mechanisms [5]. Several studies have demonstrated that individuals with a history of ACEs are more likely to develop IBS and experience greater severity of GI and extraintestinal symptoms [6]. ACEs are psychosocial stressors that intersect with social determinants of health (SDoH). SDoH comprise non-medical factors that affect health outcomes, including the conditions in which people are born and grow (pertinent to childhood), work and age (pertinent to adulthood), as well as the wider systems shaping these conditions (e.g. policies) [7]. ACEs include childhood poverty/food insecurity and various forms of household dysfunction such as neglect, incarceration, and violence [8]. Childhood poverty and food insecurity have long-lasting effects on health [9], and the family system is a powerful social context affecting long-term health [10]. Thus, ACEs may be considered as adverse SDoH [11].

The gut microbiome and its metabolites play a crucial role in the pathophysiology of IBS, particularly in individuals with a history of ACEs. ACEs can lead to long-lasting alterations in gut microbiota composition and diversity, disrupting intestinal homeostasis. One mechanism involves early life stress activating nerve growth factor (NGF)-TrkA signaling (neurotrophic tyrosine kinase receptor type 1), which increases serotonin (5-HT) production and contributes to visceral hyperalgesia [12]. Given that gut-microbiota interact with key neurochemical messengers such as 5-HT, produced both in neural cells and the GI system [13], these alterations may drive symptoms in IBS. High levels of ACEs may also impact intestinal tissue integrity [14]. Sex differences further contribute to distinct microbiome profiles in IBS, with variations in genus-level bacteria in women compared to men with IBS [15, 16]. The concept of “microgenderome” suggests that sex hormones influence gut microbial composition and function, contributing to the pathophysiology of IBS [4]. These findings collectively highlight the need for personalized microbiome-targeted approaches in managing IBS, particularly in high-risk female patients with a history of ACE.

Neuroimaging studies have provided valuable insights into brain-gut microbiome (BGM) alterations in IBS patients with a history of ACEs [17]. ACEs can lead to long-lasting changes in brain structure and function, particularly in regions involved in the stress response and emotional regulation, such as the prefrontal cortex, amygdala, and hippocampus [18]. Sex-specific differences have also been observed, with women showing decreased centrality (measure of how connected and influential a brain region is within a network) in salience and emotion regulation regions, while men exhibit increased salience network (SAL) centrality and segregation, changes linked to higher somatization [19], suggesting distinct sex-related differences in the neurodevelopmental consequences to ACEs. Women with IBS have demonstrated increased axonal strength and myelination within and between pain and sensory processing circuits, particularly in sensorimotor, corticothalamic, and basal ganglia pathways [20]. These neurobiological adaptations correlated with heightened IBS symptom severity, somatic awareness, and sensory sensitivity. Compared to men with IBS, women with IBS have exhibited greater activation in the ventromedial prefrontal cortex, right anterior cingulate cortex, and left amygdala in response to visceral stimuli [21]. This suggests that sex differences in the salience, sensorimotor, and emotional-arousal brain networks among IBS patients, potentially contributes to the heightened visceral sensitivity and altered pain perception in female IBS patients [22].

Given the clinical predominance and associated microbiome and neuroimaging changes in female IBS patients, this study aimed to identify a multi-omic (clinical, brain, gut microbiome) signature based on ACE exposure in female IBS patients compared to female HCs. Considering the role of ACEs in influencing the microbiome and brain, we hypothesized that compared to healthy females, females with IBS and a high number of ACEs would show the following: (1) greater symptoms such as GI-symptom related symptom severity, anxiety, worse, quality of life, and higher perceived stress levels, (2) gut dysbiosis as evidenced by altered bacterial metagenomics; (3) brain morphometry and connectivity signatures, reflecting regions important for stress and emotional regulation, sensory processing, and autonomic control. Unlike prior studies, this is the first study to examine clinical and behavioral outcomes in the context of gut microbial profiles and brain network connectivity, enabling a systems-level understanding of how early life adversity may shape the gut-brain axis in IBS.

## Methods

### Participant inclusion and enrollment

Premenopausal female participants with IBS or HCs were recruited by the G Oppenheimer Center for Neurobiology of Stress and Resilience at University of California Los Angeles (UCLA). All IBS patients were evaluated by a gastroenterologist or nurse practitioner with expertise in IBS for presence of a Rome IV diagnosis of IBS [23]. Rome IV diagnostic criteria for IBS is the current international standard for symptom-based diagnosis of IBS, developed by a multinational expert consensus including. The criteria require the presence of recurrent abdominal pain, on average, at least 1 day per week in the last 3 months, associated with at least two of the following: pain related to defecation, associated with a change in stool frequency, or associated with a change in stool form. The symptom onset must have occurred at least 6 months before diagnosis. Participants were excluded for the following reasons: pregnant or lactating, substance use, abdominal surgery, tobacco dependence (half a pack or more daily), extreme strenuous exercise (> 8 h of continuous exercise per week such as marathon runners), and major medical or neurological conditions. Participants taking medications that interfere with the central nervous system (unless on a steady dose for more than 3 months) or regular use of analgesic drugs were excluded. To avoid potential cofounders in microbiome analyses, included participants were required to not have taken antibiotics for at least 3 months and probiotics for at least 1 month prior to enrolling in the study. Only premenopausal females were enrolled and were scanned during the follicular phase of their menstrual cycles as determined by the self-report of their last day of the cycle. No participants exceeded 400lbs due to MRI scanning weight limits.

Participants underwent multimodal brain-imaging studies at UCLA and provided fresh stool samples for 16 s ribosomal RNA gene sequencing collected within a week of the scan. All procedures complied with the principles of the UCLA Institutional Review Board and informed consent was obtained from all participants.

### Questionnaires

Multiple validated questionnaires were used to measure baseline clinical and behavioral characteristics. We utilized multiple complementary constructs, including measures of general health, somatic symptoms, visceral sensitivity mood, anxiety, stress, and IBS-related symptoms and quality of life, as relevant to a multidimensional analysis of IBS and ACE exposure.

#### Adverse childhood experiences (ACE) questionnaire

We identified an ACE score with the 18-item expanded ACE questionnaire, which is a retrospective, self-reported, dichotomous measure designed to assess a broad spectrum of adverse experiences during childhood up to 18 years of age [8]. The cumulative score represents the overall burden of childhood adversity. It consists of two main subscales including childhood abuse and household dysfunction. It consists of three categories of childhood abuse: emotional abuse, physical abuse, and sexual abuse. It also measures four categories of exposure to household dysfunction including: substance abuse, mental illness in household, parent treated violently, parental separation or divorce, and incarcerated household member [8]. While trauma exposure assessed by the ACE Questionnaire comprise SDoH, it is not a general assessment of SDoH. The original 10-item ACE questionnaire by Felitti et al. [8], covers 10 categories of adversity which has demonstrated strong psychometric properties with a high internal consistency, with a Cronbach’s alpha of 0.88, indicating good reliability [8]. The questionnaire has also shown good test-retest reliability, with stability coefficients over time being modest to high (e.g., r=0.71). Confirmatory factor analysis has supported a two-factor structure, with factors representing child maltreatment and household dysfunction, which are highly correlated [24]. This structure has been validated in various populations, including adolescents and parent, confirming its utility across different demographic groups [24]. Our expanded questionnaire includes 18 primary questions with several sub-questions but reduces them into 8 scored categories. The score range was 0 to 8. The expanded version is helpful in clinical and research settings because it includes frequency scales and adds contextual detail to capture severity and characteristics of trauma, not just presence/absence. Both questionnaires include the same core domains listed above except for emotional and physical neglect. Overall, the ACE questionnaire is a robust instrument for evaluating childhood adversities and their long-term effects on health, offering a comprehensive measure applicable in both clinical practice and research contexts. In the present study, high ACE was defined as an ACE score of at least 2, as several studies have shown increased health risks in individuals with 2 or more ACEs [25, 26].

#### Hospital anxiety and depression scale (HADS)

The HADS is a 14-item self-report questionnaire designed to assess anxiety (HADS-A) and depression (HADS-D) without including somatic symptoms, making it particularly suitable for medically ill populations, including IBS patients, as it minimizes the risk of symptoms overlap [27]. It has good internal consistency ($\alpha$ = 0.83 for anxiety and $\alpha$ = 0.82 for depression) and has been validated for various populations [28]. The HADS shows good validity in IBS patients, with the depression subscale (HADS-D) demonstrating high correlation with other depression measures like the PHQ-9, suggesting it effectively captures depressive symptoms specific to IBS [27]. The recommended cutoff score $\ge$ 8 indicating possible cases of anxiety or depression and $\ge$ 11 indicating probable cases [27]. Higher scores on the HADS-D subscale are associated with greater psychological distress and poorer health-related quality of life, highlighting its utility in identifying comorbid anxiety and depression that may exacerbate gastrointestinal symptoms [29]. The HADS scores were used as continuous variables in the present study.

#### 12-item short form health survey (SF-12)

The SF-12 is a validated tool for capturing physical and mental health dimensions relevant to IBS patients [30]. It is effective in distinguishing impairments in IBS patients compared to the general population and other chronic diseases, particularly in areas such as energy/fatigue, role limitations due to physical health, and bodily pain [30]. The SF-12 demonstrates robust validity and reliability in IBS populations, providing a comprehensive measure of the significant impact of IBS on functional status and fell being [30]. SF-12 physical and mental health scores were used as continuous variables in the present study.

#### IBS quality of life (IBS-QOL) questionnaire

The IBS-QOL is a validated, disease-specific questionnaire designed to measure health-related quality of life (HRQOL) in patients with IBS, assessing eight domains relevant to IBS patients, including dysphoria, interference with activities, body image, health worry, food avoidance, social reactions, sexual health, and effect on relationships [31]. It demonstrates excellent psychometric properties, including internal consistency, construct validity, and responsiveness to clinical changes, making it a reliable tool for assessing the impact of IBS on quality of life and detecting changes due to therapeutic interventions [32]. Higher scores indicate better quality of life, with scores ranging from 0 to 100; a lower score suggests a greater negative impact of IBS symptoms on the patient’s quality of life, which a higher score indicates better overall well-being and fewer limitations in daily life [33]. Absolute cut-off values to categorize patients as having "good" or "poor" quality of life have not been validated or recommended in the medical literature. Instead, the IBS-QOL is primarily used to assess changes over time or in response to interventions, rather than to dichotomize patients at a specific score. The IBS-QOL score was used as a continuous variable in the present study.

#### Visceral sensitivity index (VSI)

The VSI is a 15-item self-reported scale validated in adult IBS patients to measure GI symptom-specific anxiety (GSA) [34]. It encompasses the cognitive, affective, and behavioral responses to fear of gastrointestinal sensations, symptoms, and related contexts, particularly in IBS patients [35, 36]. The VSI has demonstrated strong psychometric properties, including good internal consistency, reliability, and validity, and it effectively distinguishes between IBS patients, non-patients, and HCs while correlating with symptom severity and health-related quality of life (HRQOL) [35, 36]. Higher VSI scores, which range from 0 to 75, are associated with increased gastrointestinal symptom severity and poorer physical symptoms in IBS patients, suggesting that GI-specific anxiety is a critical mediator of IBS symptom presentation and persistence [35, 36]. It shows utility not only in understanding anxiety’s role in symptom severity and persistence but also as a potential outcome measure in therapeutic interventions [35, 36]. The VSI was used as a continuous variable in the present study.

#### Perceived stress scale (PSS)

The PSS measures the degree to which individuals perceive situations in their lives as stressful, capturing the central components of stress experience, including feelings of unpredictability, uncontrollability, and overload [37]. In IBS patients, the PSS is a valid and reliable measure for assessing perceived stress, showing significant correlations with psychological distress, including anxiety, depression, and visceral sensitivity, which are known to exacerbate IBS symptoms [38]. Higher PSS scores indicate greater perceived stress, which is associated with increased severity of IBS symptoms, poorer quality of life, and a heightened perception of symptom burden, emphasizing the importance of stress management in IBS treatment [38]. The PSS score was used as a continuous variable in the present study.

#### Patient health questionnaire-15 (PHQ-15)

The PHQ-15 is a self-administered questionnaire designed to measure the severity of somatic symptoms, with scores ranging from 0 to 30; higher scores indicate greater somatic symptom severity [39]. It has been validated as a reliable measure for assessing somatization in IBS patients, showing strong associations with psychological distress, functional impairment, and healthcare utilization [39]. In IBS populations, higher PHQ-15 scores are associated with increased psychological distress, including anxiety and depression, as well as greater severity of gastrointestinal symptoms and reduced quality of life [29]. It categorizes somatic symptom severity as minimal (0–4), low (5–9), medium (10–14), and high (15–30), with scores of 15 or higher indicating clinically significant somatic symptom severity that may require psychological intervention [39]. The PHQ-15 score was used as a continuous variable in the present study [40].

#### Irritable bowel syndrome severity scoring system (IBS-SSS)

The IBS-SSS is a quantitative tool designed to assess IBS severity based on five key symptom domains: abdominal pain intensity, abdominal pain frequency, distension, stool frequency and consistency, and quality of life interference. Each of the 5 items are scored on a visual analog scale from 0 to 100 and the total score ranges from 0 to 500, with severity categorized as mild (<175), moderate (175–300), or severe ($\ge$ 300), allowing for standardized assessment of symptom burden and treatment response. The scale demonstrated high reliability, construct validity, and sensitivity to change, with a $\ge$ 50-point reduction considered clinically meaningful for symptom improvement. Given its simplicity and responsiveness, the IBS-SSS is a validated and widely used tool in both clinical and research settings. The IBS-SSS was used as a continuous variable in the present study.

### Gut microbiome

Participants’ stool samples were collected using standardized procedures to ensure sample integrity and minimize contamination. To avoid cross-contamination, participants were instructed to collect the sample without allowing contact with urine or toilet water. Following collection, the samples were immediately sealed, labeled with the participant’s unique study identifier, and stored at 4 °C until transportation to the laboratory. Samples were processed within 24 hours of collection, aliquoted into sterile cryovials, and stored at −80°C for subsequent microbiome analyses.

DNA from stool was extracted using the DNA Fecal Microbe Miniprep Kit (Zymo Research). The V4 region of 16S ribosomal RNA was amplified and underwent paired end sequencing on an Illumina HiSeq 2500. Sequences were processed through the DADA2 pipeline to generate exact amplicon sequence variants (ASVs) and taxonomy was assigned based upon the SILVA 138 database. Predicted metagenomics was performed using PICRUSt2 in QIIME2 with the default settings to predict abundances of bacterial gene families annotated as KEGG orthologs (KO) based on nearest reference genomes to 16S sequences.

### Structural and functional brain imaging

*Acquisition:* Whole brain structural and resting-state scans were acquired at the Ahmanson-Lovelace Brain Mapping Center on a 3.0 Tesla Siemens Prisma MRI Scanner (Siemens, Erlangen, Germany). Comprehensive information on the standardized acquisition protocols, quality control measures, and image preprocessing can be found in previously published studies [41–43]. High-resolution T1-weighted images were obtained with the following parameters: echo time/repetition time (TE/TR) = 3.26 ms/2200 ms, field of view = 220 × 220 mm, slice thickness = 1 mm, 176 slices, a 256 × 256 voxel matrix, and a voxel size of 0.86 × 0.86 × 1 mm. Whole-brain resting-state images were acquired with participants’ eyes closed using an echo-planar sequence with the following parameters: echo time/repetition time (TE/TR) = 28 ms/2000 ms, flip angle = 77°, scan duration = 10 minutes 6 seconds, field of view (FOV) = 220 mm, 40 slices, and a slice thickness of 4.0 mm. A diffusion weighted image was acquired to assess white matter anatomical connectivity (64 noncollinear directions, b = 1000 s/mm^2^, 9 b = 0 s/mm^2^ images, TR: 9500ms, TE: 88ms, field of view: 2304 x 2304, acquisition matrix: 128 x 128, slice thickness: 2 mm, spacing between slices: 2 mm).

*Structural Processing:* Cortical reconstruction and volumetric segmentation was done using FreeSurfer 7 [44]. FreeSurfer-processed structural images were parcellated using the Destrieux cortical atlas [45], Harvard-Oxford subcortical atlas [46] and Ascending Arousal atlas [47–49], and values of cortical thickness, surface area, mean curvature and volume for cortical regions, and volume for subcortical regions, were extracted.

*Functional Image Processing.* All functional images were preprocessed using a pipeline for volume-based resting-state functional connectivity (rs-FC) analyses in CONN [50]. All functional images underwent realignment and unwarping, slice-timing correction, and ART-based identification of outlier scans for scrubbing. Using structural image data, functional images were normalized and segmented into grey matter, white matter, and CSF tissue [51]. Functional images were then denoised by using ordinary least squares (OLS) regression of potential confounders and temporal band-pass filtering. Specifically, we used the default anatomical component-based noise correction procedure (aCompCor), which includes noise components from white-matter, cerebrospinal fluid [52], estimated subject-motion parameters [53], scrubbing of outlier scans based on framewise displacement [54], and removal of potential ramping effects at the start of the session [55]. A temporal band-pass filter of 0.008–0.09 Hz after regression was used to minimize the influence of physiological and head motion, and other noise sources [56]. Fisher-transformed correlations (Z) between the functional time series of each pair of regions, for all parcellated regions, were computed in CONN to derive a 165 x 165 matrix for each participant. The bottom half of the undirected matrix was then concatenated into one vector for each participant, representing the resting-state functional connectivity between every ROI pair.

*Diffusion Processing:* All diffusion weighted images were corrected using DiPy’s Median Otsu algorithm for skull stripping and denoising. Each scan underwent translation, rigid, and affine registration using the aforementioned atlases. The resulting homography was also used to transform the b-vectors and b-values. Finally, diffusion tensors were fit, and fractional anisotropy was calculated. This process resulted in a 188x76 matrix, with each row representing a participant and each column representing a diffusion feature.

### Statistical analyses

To capture the complex relationship between ACEs and IBS, the model integrated multiple omics and behavioral assessments through Stacked Ensemble Modeling [57–59], with Extreme Gradient Boosting (XGBoost) as base models and a Support Vector Machine (SVM) as the meta-learner. Given the variability in range and dimensionality across the 5 datasets, hyperparameters such as L1 and L2 regularization were optimized for each omic. Regularization penalized redundant or weak predictors, enhancing interpretability and reducing overfitting. Importantly, decision tree-based methods, including XGBoost, are inherently robust to multicollinearity because splits are determined by maximizing information gain rather than relying on linear correlations, thereby minimizing bias introduced by overlapping constructs.{Piramuthu, 2008 #4051} This approach ensured that overlapping behavioral measures did not disproportionately influence the models while retaining complementary predictive information.

### Model implementation

The data underwent 5-fold cross validation. Each iteration involved an 80% training set (n=150 or 151) and a 20% testing set (n=37 or 38). Within each training fold, the data was further partitioned into five sub-folds to facilitate meta-model training and hyperparameter optimization. During the training of each base model, optimal training parameters were identified through an iterative tuning process, ensuring the selection of parameters that maximized model performance prior to their application in the final base models.

### Data preparation

Each data block was examined for response variables that could disrupt the analysis. From the clinical dataset, columns ACE Total Score and Group (IBS or HC) were removed as they were directly related to the response variable. IBS-QOL and IBS-SSS metrics were only measured for participants labeled as IBS and were imputed with a 0 for HCs. For the metagenomic dataset, to mitigate skewed distributions of bacterial counts, log transformation was applied, and outputs were then z-normalized across subjects for each bacterial strain. Through the base-model training step of the architecture, the features were reduced in the following way: 52 clinical variables down to 50, 2661 metagenomic variables down to 183, 658 structural brain features down to 168, 76 diffusion fractional anisotropy (dti.fa) features down to 60, and 15753 resting-state functional features (rspw) down to 111.

### Evaluation and interpretation methods

Gain analysis was used to identify the features that contributed the most to the model’s loss reduction and were visualized through bar plots. To further elucidate the associations between each high-gain variable and subject classes, SHAP and SHAP dependence analysis were utilized. Associations visualized through tile plots, highlighting the contribution of selected features to each subject class [60]. To report significant pairwise Pearson correlations between each data modality, connectograms were used to depict the relationships between features with correlation r >=0.4. For reader interpretability purposes, only the top variables responsible for 30% of each base model’s gain are provided in all visualizations.

To assess the predictive performance of our model, we report the Cohen’s kappa coefficient as a measure of agreement between predicted and actual classifications [61]. Kappa values serve as more interpretable alternatives to error rate in the context of multiclass classification, though for further interpretability, simple accuracies were also calculated and reported.

We also utilized the Receiver Operating Characteristic (ROC) curve and the Area Under the Curve (AUC) metric. Since our task involved multiclass classification, we employed a one-vs-rest (OvR) approach, where an ROC curve was generated for each class against the remaining classes. An ROC curve was also generated for the meta model.

### Clinical and behavioral differences

To identify clinical measures that demonstrated statistically significant differences between groups, analyses of variance (ANOVA) and chi-squared tests were applied. To further elucidate between-group differences, results of pairwise linear contrasts were also calculated.

## Results

### Participant demographics and clinical measures

Participant characteristics are provided in Tables 1 and 2. In total, 188 premenopausal female participants (mean age=30 years old) were classified into four subgroups based on IBS diagnosis and ACE Total score, with high ACE defined as ≥2 and low ACE as 0–1: IBS with high ACE (n=37); IBS with low ACE (n=55); HCs with high ACE (n=19); and HCs with low ACE (n=77).

**Table 1:** Participant characteristics

Variable	HC, Low ACE, n = 77			HC, High ACE, n = 19			IBS, Low ACE, n = 55			IBS, High ACE, n = 37			All Class, n = 188		
	Mean	SD	Range	Mean	SD	Range	Mean	SD	Range	Mean	SD	Range	Mean	SD	Range
Age	30.49	12.26	[18, 58]	29.79	11.76	[18, 60]	28.76	10.16	[18, 59]	33.30	10.18	[18, 60]	30.47	11.25	[18, 60]
Bowel Habits (BH)															
Weight	155.27	32.77	[102,234]	158.79	32.54	[109,209.39]	148.92	32.07	[105,239]	147.16	25.71	[95,196.80]	152.20	31.28	[95,239]
Adverse Childhood Experience (ACE) Questionnaire															
Emotional Abuse	0.01	0.11	[0,1]	0.53	0.51	[0,1]	0.04	0.19	[0,1]	0.30	0.46	[0,1]	0.13	0.33	[0,1]
Physical Abuse	0.00	0.00	[0,0]	0.21	0.42	[0,1]	0.00	0.00	[0,0]	0.08	0.28	[0,1]	0.04	0.19	[0,1]
Sexual Abuse	0.04	0.19	[0,1]	0.11	0.32	[0,1]	0.07	0.26	[0,1]	0.24	0.43	[0,1]	0.10	0.30	[0,1]
Substance Abuse	0.06	0.25	[0,1]	0.53	0.51	[0,1]	0.05	0.23	[0,1]	0.43	0.50	[0,1]	0.18	0.39	[0,1]
Parental Divorce/Separation	0.19	0.40	[0,1]	0.63	0.50	[0,1]	0.11	0.31	[0,1]	0.68	0.47	[0,1]	0.31	0.46	[0,1]
Household Mental Illness	0.04	0.19	[0,1]	0.63	0.50	[0,1]	0.18	0.39	[0,1]	0.65	0.48	[0,1]	0.26	0.44	[0,1]
Incarcerated Household Member	0.01	0.11	[0,1]	0.11	0.32	[0,1]	0.02	0.13	[0,1]	0.08	0.28	[0,1]	0.04	0.19	[0,1]
Parents Treated Violently	0.01	0.11	[0,1]	0.11	0.32	[0,1]	0.00	0.00	[0,0]	0.24	0.43	[0,1]	0.06	0.25	[0,1]
Hospital Anxiety and Depression (HAD) Questionnaire															
Anxiety	3.48	2.71	[0,10]	4.11	3.28	[0,13]	7.44	4.42	[0,18]	6.57	3.63	[1, 16]	5.33	3.91	[0,18]
Depression	1.52	2.05	[0,11]	1.32	1.73	[0,7]	3.11	3.22	[0,16]	3.03	2.40	[0,9]	2.26	2.60	[0,16]
HAD Total	5.02	4.26	[0,21]	5.42	4.29	[0,16]	10.55	6.56	[0,34]	9.59	5.22	[2, 23]	7.58	5.78	[0,34]
SF12 Questionnaire															
SF12 PCS	54.83	4.93	[32.10,64.83]	54.98	2.57	[46.48,57.94]	52.26	5.26	[34.26,61.37]	50.07	7.21	[28.33,61.54]	53.18	5.66	[28.33,64.83]
SF12 MCS	53.02	6.06	[34.13,60.91]	53.05	5.39	[38.481,59.83]	47.49	8.45	[28.33,62.03]	46.04	9.27	[23.20,60.77]	50.06	8.02	[23.20,62.03]
Irritable Bowel Syndrome Quality of Life (IBSQoL) Questionnaire															
Dysphoria							16.13	6.86	[8, 35]	18.35	9.07	[0,39]	8.33	10.14	[0,39]
Interference							15.85	6.63	[7, 32]	15.65	7.52	[0,35]	7.72	9.28	[0,35]
Body Image							8.71	3.41	[4, 18]	8.92	4.05	[0,19]	4.30	5.09	[0,19]
Health Worry							7.00	2.82	[3, 14]	6.86	3.42	[0,14]	3.40	4.08	[0,14]
Food Avoidance							8.38	3.30	[3, 15]	8.24	4.21	[0,15]	4.07	4.90	[0,15]
Social Reaction							8.24	3.32	[4, 15]	9.05	4.44	[0,19]	4.19	5.05	[0,19]
Sexual Concerns							3.69	2.32	[0,10]	4.22	2.00	[0,10]	1.91	2.49	[0,10]
Relationship							5.42	2.23	[3, 13]	5.68	3.17	[0,15]	2.70	3.32	[0,15]
Total							73.42	24.82	[34,136]	75.22	34.57	[0,151]	36.28	42.30	[0,151]
Other Measures															
VSI Score	5.40	10.33	[0,75]	2.00	3.25	[0,12]	32.36	14.97	[2, 65]	35.41	14.12	[7, 65]	19.00	18.89	[0,75]
PSS Score	10.41	5.69	[1, 30]	12.00	6.70	[0,26]	15.76	5.79	[0,29]	15.54	6.79	[4, 29]	13.16	6.50	[0,30]
PHQ Score	1.71	1.83	[0,9]	3.68	2.43	[0,9]	9.80	3.64	[3, 19]	10.68	3.80	[4, 18]	6.07	5.00	[0,19]
IBS Symptom Severity							205.89	91.14	[0,429]	210.46	74.65	[0,338]	101.65	119.64	[0,429]

HC: Healthy Control. IBS: Irritable Bowel Syndrome.

N=188 total, HC low ACE group n=77, HC high ACE group n=119, IBS low ACE group n=55, IBS high ACE group n=37.Means and standard deviations (SD) are reported for continuous variables.

For categorical variables, Means and standard deviations (SD) are reported as NA and 0, respectively.

AL: ACE Low. AH: ACE High.

BH: Bowel Habits. ACE: Adverse Childhood Effects. SF12_PCS: The Physical Component Summary score of the Short Form-12 Health Survey. SF12_MCS: The Mental Component Summary Score of the Short Form-12 Health Survey. IBSQoL: Irritable Bowel Syndrome Quality of Life. VSI: Visceral Sensitivity Index. PSS Score: Perceived Stress Scale. PHQ Score: Patient Health Questionnaire Score

**Table 2:** Group differences in clinical and behavioral variables

Variable	ANOVA/ChiSq	HC vs IBS		AL vs AH		IBS AL vs IBS AH		HC AL vs HC AH		HC AH vs IBS AH		HC AL vs IBS AL	
	p-val	t-val	p-val	t-val	p-val	t-val	p-val	t-val	p-val	t-val	p-val	t-val	p-val
Age	0.30	−0.48	0.64	−1.02	0.31	−1.90	0.06	0.24	0.81	−1.11	0.27	0.87	0.38
Bowel Habits (BH)	2E-16												
Weight	0.40	1.68	0.10	−0.16	0.87	0.26	0.80	−0.43	0.67	1.27	0.20	1.13	0.26
Adverse Childhood Experience (ACE) Questionnaire													
Emotional Abuse	2E-16	2.14	0.03	−8.04	2E-16	−4.24	2E-16	−6.93	2E-16	2.81	0.01	−0.46	0.65
Physical Abuse	2E-16	2.17	0.03	−4.89	2E-16	−2.13	0.03	−4.58	2E-16	2.56	0.01	0.00	1.00
Sexual Abuse	0.01	−1.80	0.07	−2.48	0.01	−2.79	0.01	−0.90	0.37	−1.70	0.09	−0.67	0.51
Substance Abuse	2E-16	0.92	0.36	−7.40	2E-16	−5.22	2E-16	−5.29	2E-16	0.98	0.33	0.17	0.86
Parental Divorce/Separation	2E-16	0.31	0.76	−7.47	2E-16	−6.60	2E-16	−4.23	2E-16	−0.39	0.70	1.20	0.23
Household Mental Illness	2E-16	−1.33	0.18	−8.83	2E-16	−6.09	2E-16	−6.42	2E-16	−0.17	0.87	−2.24	0.03
Incarcerated Household Member	0.10	0.30	0.76	−2.48	0.01	−1.57	0.12	−1.91	0.06	0.46	0.65	−0.16	0.88
Parents Treated Violently	2E-16	−1.64	0.10	−4.42	2E-16	−5.01	2E-16	−1.58	0.12	−2.14	0.03	0.32	0.75
Hospital Anxiety and Depression (HAD) Questionnaire													
Anxiety	2E-16	−5.44	2E-16	0.21	0.84	1.15	0.25	−0.69	0.49	−2.46	0.01	−6.29	2E-16
Depression	2E-16	−3.98	2E-16	0.34	0.73	0.16	0.88	0.32	0.75	−2.44	0.02	−3.62	2E-16
HAD Total	2E-16	−5.58	2E-16	0.32	0.75	0.86	0.39	−0.30	0.77	−2.83	0.01	−5.99	2E-16
SF12 Questionnaire													
SF12 PCS	2E-16	4.13	2E-16	1.13	0.26	1.88	0.06	−0.11	0.91	3.20	2E-16	2.67	0.01
SF12 MCS	2E-16	4.99	2E-16	0.56	0.57	0.89	0.37	−0.01	0.99	3.29	2E-16	4.15	2E-16
Irritable Bowel Syndrome Quality of Life (IBSQoL) Questionnaire													
Dysphoria				−1.22	0.22	−1.91	0.06						
Interference				0.13	0.90	0.20	0.84						
Body Image				−0.24	0.81	−0.38	0.70						
Health Worry				0.19	0.85	0.30	0.77						
Food Avoidance				0.16	0.87	0.25	0.80						
Social Reaction				−0.92	0.36	−1.44	0.15						
Sexual Concerns				−1.03	0.31	−1.61	0.11						
Relationship				−0.42	0.68	−0.65	0.51						
Total				−0.27	0.79	−0.42	0.68						
Other Measures													
VSI Score	2E-16	−14.71	2E-16	0.09	0.93	−1.16	0.25	1.08	0.28	−9.62	2E-16	−12.34	2E-16
PSS Score	2E-16	−4.36	2E-16	−0.67	0.50	0.17	0.86	−1.01	0.31	−2.05	0.04	−4.91	2E-16
PHQ Score	2E-16	−15.21	2E-16	−2.87	2E-16	−1.38	0.17	−2.59	0.01	−8.34	2E-16	−15.27	2E-16
IBS Symptom Severity	2E-16			−0.23	0.82	−0.36	0.71						

HC: Healthy Control. IBS: Irritable Bowel Syndrome

N = 188 total, HC low ACE group n = 77, HC high ACE group n = 119, IBS low ACE group n = 55, IBS high ACE group n = 37

Means and standard deviations (SD) are reported for continuous variables

For categorical variables, Means and standard deviations (SD) are reported as NA and 0, respectively

AL: ACE Low. AH: ACE High

BH: Bowel Habits. ACE: Adverse Childhood Effects. SF12_PCS: The Physical Component Summary score of the Short Form-12 Health Survey. SF12_MCS: The Mental Component Summary Score of the Short Form-12 Health Survey. IBSQoL: Irritable Bowel Syndrome Quality of Life. VSI: Visceral Sensitivity Index. PSS Score: Perceived Stress Scale. PHQ Score: Patient Health Questionnaire Score

#### IBS disease-related differences

There were significant differences in ACE subscale scores between IBS patients and HCs (emotional abuse: p=.03, physical abuse: p=.03). In addition, IBS patients had worse scores on current anxiety (p $=$ 2x10^−16^) and depression (p $=$ 2x10^−16^) symptoms, physical (PCS) and mental (MCS) quality of life (p’s $=$ 2x10^−16^), GI symptom-related anxiety (VSI) (p $=$ 2x10^−16^), perceived stress levels (PSS) (p $=$.2x10^−16^), and somatic symptom severity (PHQ-15) (p $=$ 2x10^−16^).

#### Adverse childhood-related differences

All individuals with high ACE total scores compared to low ACE scores had worse somatic symptom severity scores (PHQ-15) (p $=$ 2x10^−16^).

There were no significant differences in symptoms between the IBS patients with high ACE scores compared to IBS patients with low ACE scores. Although IBS-SSS and IBS-QOL were worse in IBS, high ACE overall compared to IBS with low ACE, these differences were not statistically significant.

Compared to HCs with low ACE, HCs with high ACE scores reported worse somatic symptom severity scores (PHQ-15) (p=.01).

#### Disease and ACE-related differences (i.e., interaction effects)

Specifically, looking at individuals with high ACE total scores, compared to HCs, IBS patients also had higher scores on subscale of parents treated violently (p=.03), anxiety and depression symptoms (p $=$.01, P=.02), GI symptom-related anxiety (VSI) (p $=$ 2x10^−16^), perceived stress levels (PSS) (p $=$.04), poorer PCS and MCS quality of life (p’s $=$ 2x10^−16^), and worse somatic symptom severity (PHQ-15) (p $=$ 2x10^−16^).

However, in IBS and HC participants with high ACE scores, HCs had higher scores on the emotional and physical abuse subscales (p’s=.01).

### Stacked ensemble model performance

The overall model exhibited an estimated kappa of 0.67, signifying high levels of agreement between the true and predicted labels.

The overall model yielded a ROC with an AUC of 0.70 for identifying HC, high ACE participants; 0.82 for IBS, low ACE participants; and 0.87 for IBS, high ACE participants. The accuracy for all base models were also calculated at 78.19%.

### Selected variables

**Fig. 1:**
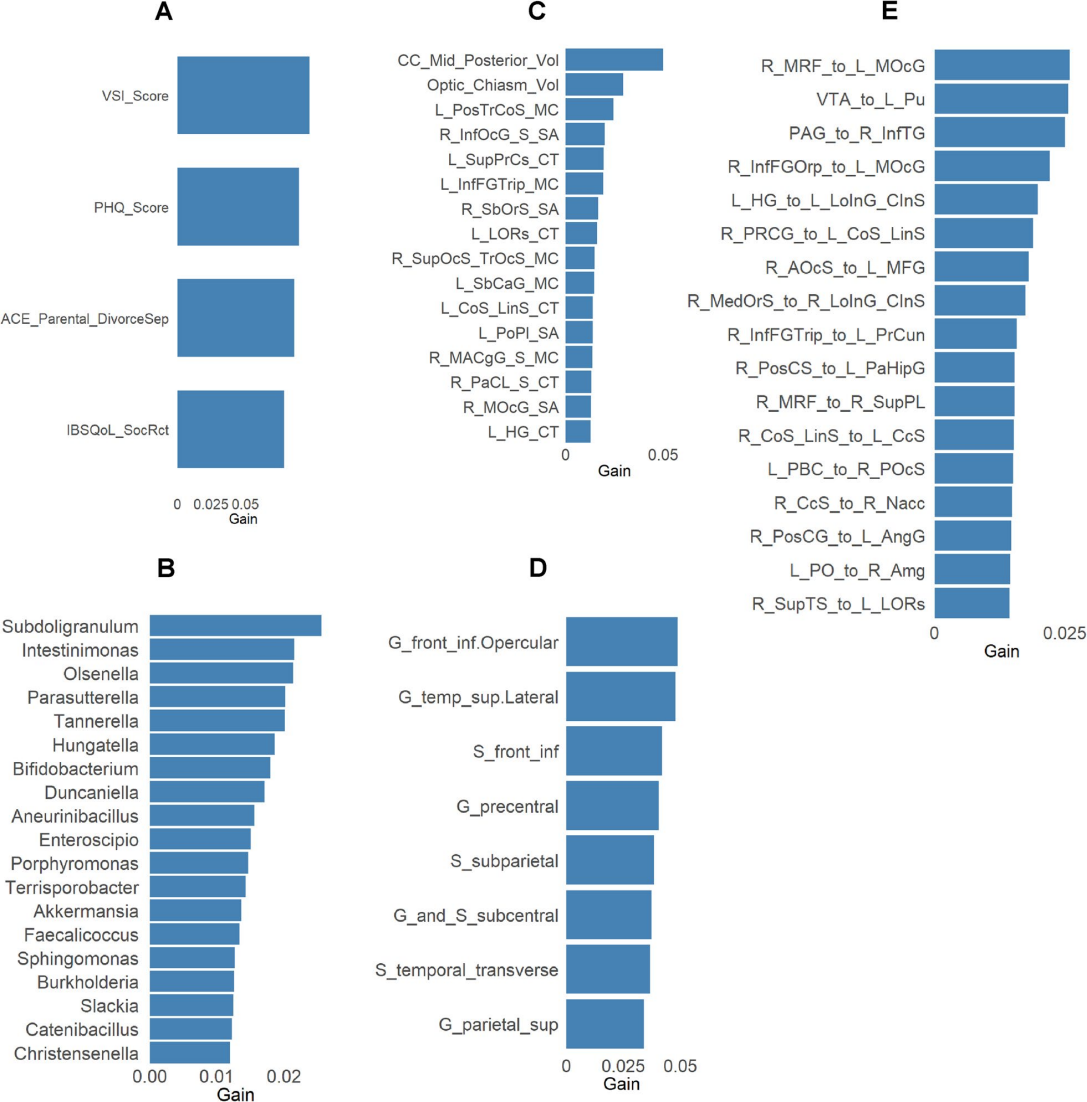
Significant features for each dataset. The highest gain features comprising 30% of total gain is reported. HC: Healthy Control. IBS: Irritable Bowel Syndrome. N = 188 total, HC low ACE group n = 77, HC high ACE group n = 119, IBS low ACE group n = 55, IBS high ACE group n = 37.AL: ACE Low. AH: ACE High. (A) Clinical Features (B) Metagenomic Features (C) Structural Brain Features (D) Diffusion Tensor Imaging Brain Features (Fractional Anisotropy) (E) Resting State Pairwise Brain Features Clinical Abbreviations: IBSQoL_DysphR: IBS Quality of Life Dysphoria. VSI_Score: Visceral Sensitivity Index. IBSQoL_SocRct: IBS Quality of Life Social Reaction. Brain region abbreviations for resting-state, diffusion tensor imaging, and structural scans are listed in Table 7

The base models of the ensemble architecture performed feature selection, yielding a total of 424 features: 50 clinical, 183 metagenomic, 168 structural MRI, 60 diffusion, and 111 resting-state connectivity features. The most significant features that cumulatively accounted for at least 30% of the model’s gain are visualized in Fig 1.

### SHAP dependence

**Fig. 2:**
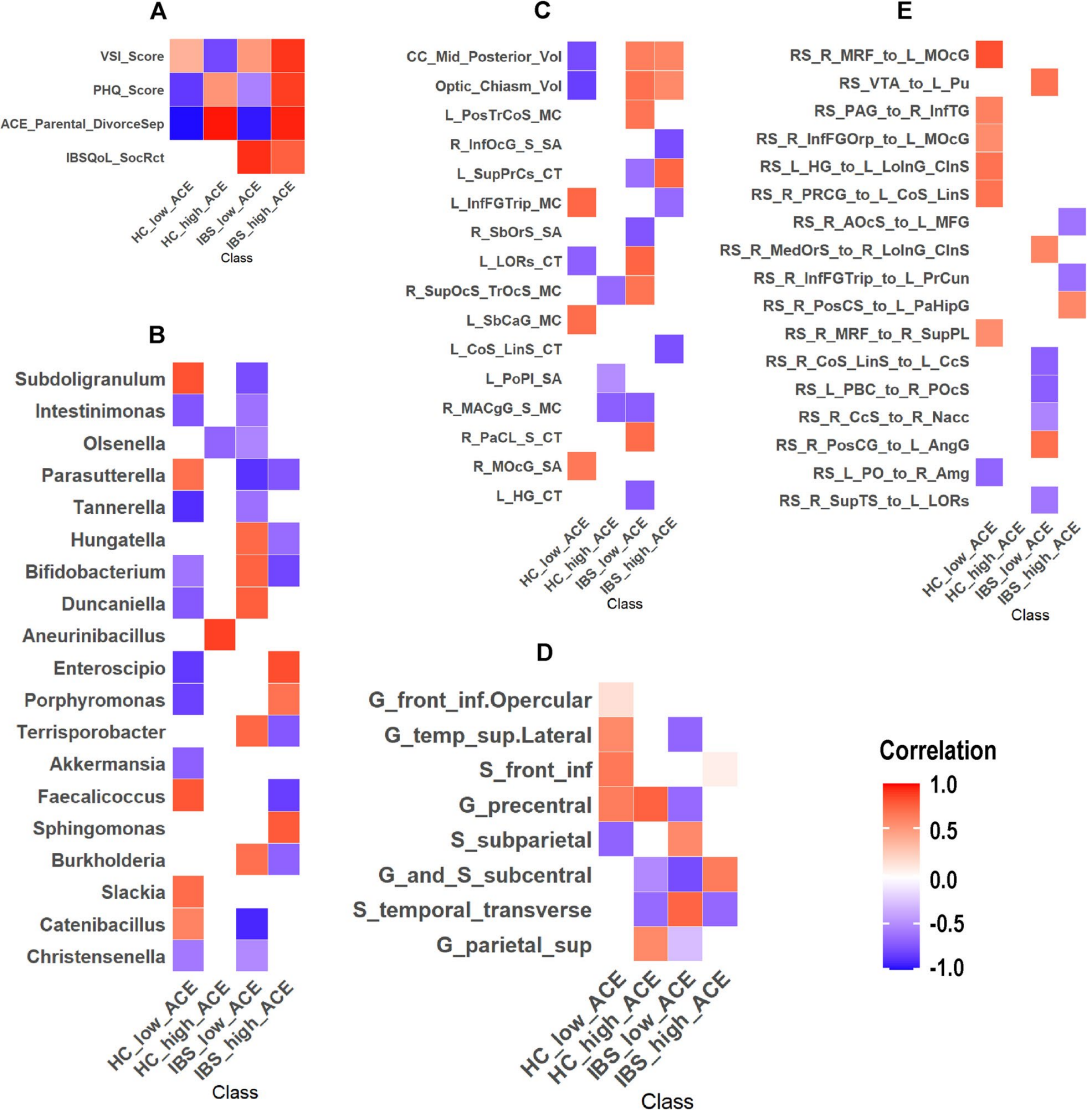
SHAP dependence plots for the most significant features of each block. HC: Healthy Control. IBS: Irritable Bowel Syndrome. N = 188 total, HC low ACE group n = 77, HC high ACE group n = 119, IBS low ACE group n = 55, IBS high ACE group n = 37. AL: ACE Low. AH: ACE High. (A) Clinical Features (B) Metagenomic Features (C) Structural Brain Features (D) Diffusion Tensor Imaging Brain Features (Fractional Anisotropy) (E) Resting State Pairwise Brain Features Clinical Abbreviations: IBSQoL_DysphR: IBS Quality of Life Dysphoria. VSI_Score: Visceral Sensitivity Index. IBSQoL_SocRct: IBS Quality of Life Social Reaction. Brain region abbreviations for resting-state, diffusion tensor imaging, and structural scans are listed in Table 7

For additional interpretability of the selected features, SHAP dependence plots (Table 3, Fig 2) were used to identify how changes in each feature influenced class predictions.

**Table 3:** Significant features from the model

Feature	Gain	Cover	Freq		Feature	Gain	Cover	Freq	Network
Clinical					Structural				
VSI Score	0.1	0.11	0.1		R_SbOrS_SA	0.02	0.01	0.01	CAN, SAL
PHQ Score	0.09	0.09	0.08		L_LORs_CT	0.02	0.02	0.01	CAN, SAL
ACE Parental DivorceSep	0.09	0.08	0.08		L_PosTrCoS_MC	0.02	0.03	0.02	DMN
IBSQoL SocRct	0.08	0.03	0.02		L_SbCaG_MC	0.01	0.01	0.01	DMN
Metagenomic					L_PoPl_SA	0.01	0.02	0.02	DMN
Subdoligranulum	0.03	0.02	0.01		L_HG_CT	0.01	0.01	0.01	DMN
Intestinimonas	0.02	0.02	0.02		L_InfFGTrip_MC	0.02	0.01	0.01	ERN
Olsenella	0.02	0.02	0.02		R_InfOcG_S_SA	0.02	0.02	0.02	OCC
Parasutterella	0.02	0.02	0.02		R_SupOcS_TrOcS_MC	0.01	0.02	0.01	OCC
Tannerella	0.02	0.01	0.02		R_MOcG_SA	0.01	0.01	0.01	OCC
Hungatella	0.02	0.02	0.02		L_CoS_LinS_CT	0.01	0.01	0.01	SAL
Bifidobacterium	0.02	0.02	0.02		R_MACgG_S_MC	0.01	0.02	0.01	SAL
Duncaniella	0.02	0.01	0.01		CC_Mid_Posterior_Vol	0.05	0.04	0.03	SMN
Aneurinibacillus	0.02	0.02	0.02		Optic_Chiasm_Vol	0.03	0.03	0.02	SMN
Enteroscipio	0.02	0.02	0.01		L_SupPrCs_CT	0.02	0.02	0.02	SMN
Porphyromonas	0.01	0.01	0.01		R_PaCL_S_CT	0.01	0.01	0.01	SMN
Terrisporobacter	0.01	0.01	0.01		Diffusion				
Akkermansia	0.01	0.01	0.01		S_subparietal	0.04	0.04	0.03	CEN
Faecalicoccus	0.01	0.01	0.01		G_parietal_sup	0.03	0.04	0.04	CEN
Sphingomonas	0.01	0.01	0.01		G_temp_sup.Lateral	0.05	0.05	0.03	DMN
Burkholderia	0.01	0.01	0.01		S_temporal_transverse	0.04	0.04	0.04	DMN
Slackia	0.01	0.01	0.01		S_front_inf	0.04	0.03	0.03	ERN
Catenibacillus	0.01	0.01	0.01		G_front_inf.Opercular	0.05	0.04	0.03	SMN
Christensenella	0.01	0.01	0.01		G_precentral	0.04	0.04	0.03	SMN
Resting-State Pairwise					G_and_S_subcentral	0.04	0.03	0.03	SMN
RS_R_MRF_to_L_MOcG	0.03	0.03	0.02						
RS_VTA_to_L_Pu	0.03	0.02	0.02						
RS_PAG_to_R_InfTG	0.02	0.01	0.01						
RS_R_InfFGOrp_to_L_MOcG	0.02	0.01	0.01						
RS_L_HG_to_L_LoInG_CInS	0.02	0.01	0.01						
RS_R_PRCG_to_L_CoS_LinS	0.02	0.01	0.01						
RS_R_AOcS_to_L_MFG	0.02	0.01	0.01						
RS_R_MedOrS_to_R_LoInG_CInS	0.02	0.01	0.01						
RS_R_InfFGTrip_to_L_PrCun	0.02	0.01	0.01						
RS_R_PosCS_to_L_PaHipG	0.02	0.01	0.01						
RS_R_MRF_to_R_SupPL	0.02	0.01	0.01						
RS_R_CoS_LinS_to_L_CcS	0.02	0.01	0.01						
RS_L_PBC_to_R_POcS	0.01	0.01	0.01						
RS_R_CcS_to_R_Nacc	0.01	0.01	0.01						
RS_R_PosCG_to_L_AngG	0.01	0.01	0.01						
RS_L_PO_to_R_Amg	0.01	0.01	0.01						
RS_R_SupTS_to_L_LORs	0.01	0	0.01						

CAN: Central Autonomic Network, SAL: Salience Network, DMN: Default Mode Network, OCC: Occipital Network, SMN: Sensorimotor Network, CEN: Central Executive Network, ERN: Emotion Regulation Vol: Volume, CT: cortical thickness MC: Mean curvature SA: Surface area

Clinical Abbreviations: IBSQoL_DysphR: IBS Quality of Life Dysphoria. VSI_Score: Visceral Sensitivity Index. IBSQoL_SocRct: IBS Quality of Life Social Reaction

Brain region abbreviations for resting-state, diffusion tensor imaging, and structural scans are listed in Table 7

In descending order of importance, the top clinical variables included were VSI Score, PHQ-15 Score, ACE Parental Divorce/Separation, and IBS QOL Social Reaction. All three high-gain variables were related to GI symptoms, and their SHAP values were strongly associated with patient classes exhibiting IBS (Table 3, Fig 2a).

Bacterial transcriptomes in order of importance are listed in Table 3, and their associations to each of the 4 groups are depicted in Fig 2b**.** For example, the most significant bacteria contributing to the model included *Akkermansia*, *Bifidobacterium, Intestinimonas, Subdoligranulum, Christensenella,* and* Burkholderia.*

Structural, diffusion, and resting-state pairwise brain signatures identified as the most significant for prediction of the 4 participants groups are listed in Table 3 in descending order. Their contributions to the prediction of each participant group are depicted in Figures 2c, 2 d, and 2e, respectively. The key brain regions contributing to the model included those from the central autonomic, (CAN), salience (SAL), sensorimotor (SMN), default mode (DMN), and emotion regulation (ERN) networks.

### Multi-omics relationships:

**Fig. 3:**
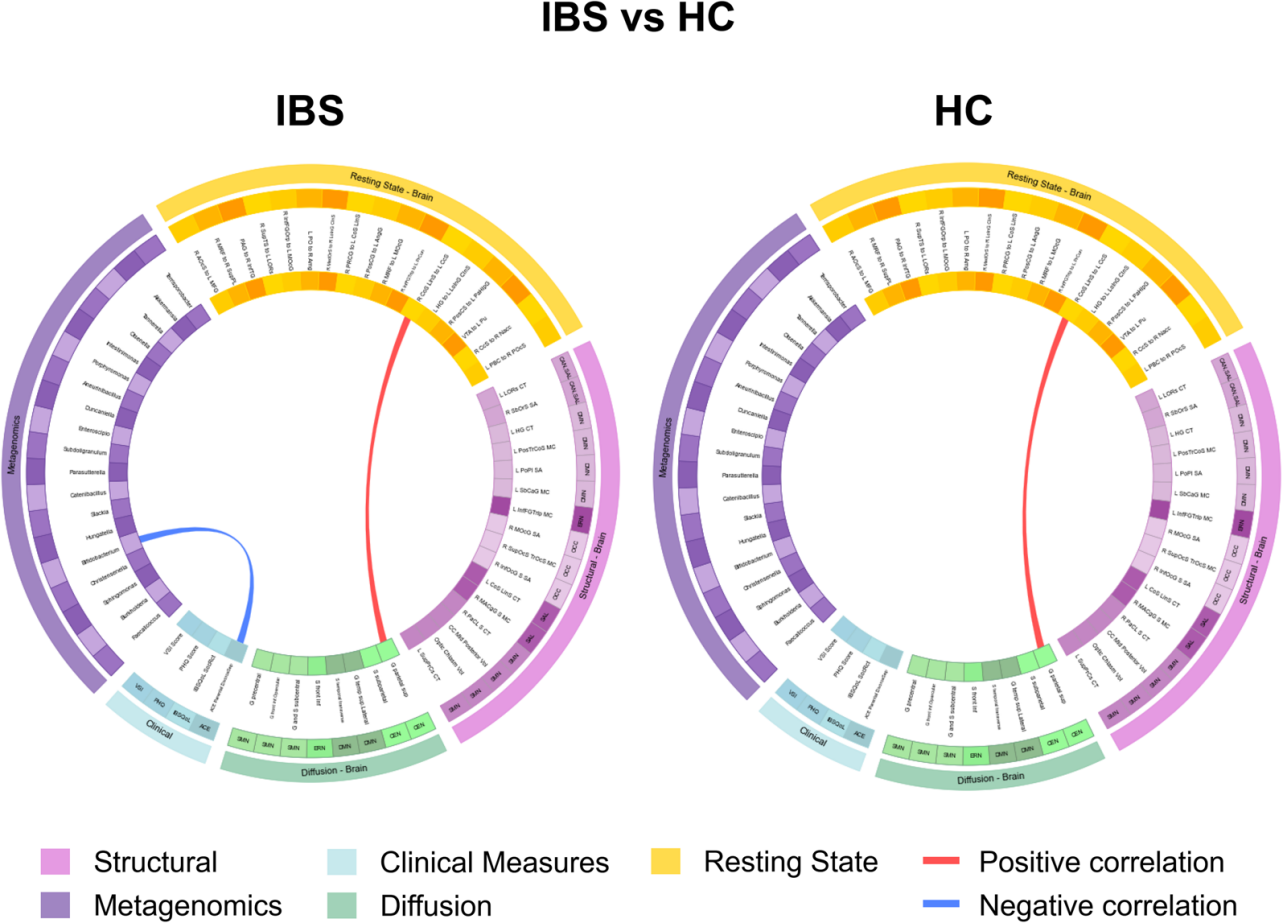
Connectograms comparing IBS vs HC.HC: Healthy Control. IBS: Irritable Bowel Syndrome. N = 188 total, HC group n = 96, IBS group n = 92 Dti_fa: diffusion tensor imaging Fractional Anisotropy, rspw: resting state pairwise, struc: structural, metag: metagenomic, clin: clinical ACE Parental DivorceSep: Adverse Childhood Experiences, Parental Divorce. Brain region abbreviations for resting-state, diffusion tensor imaging, and structural scans are listed in Table 7

**Fig. 4:**
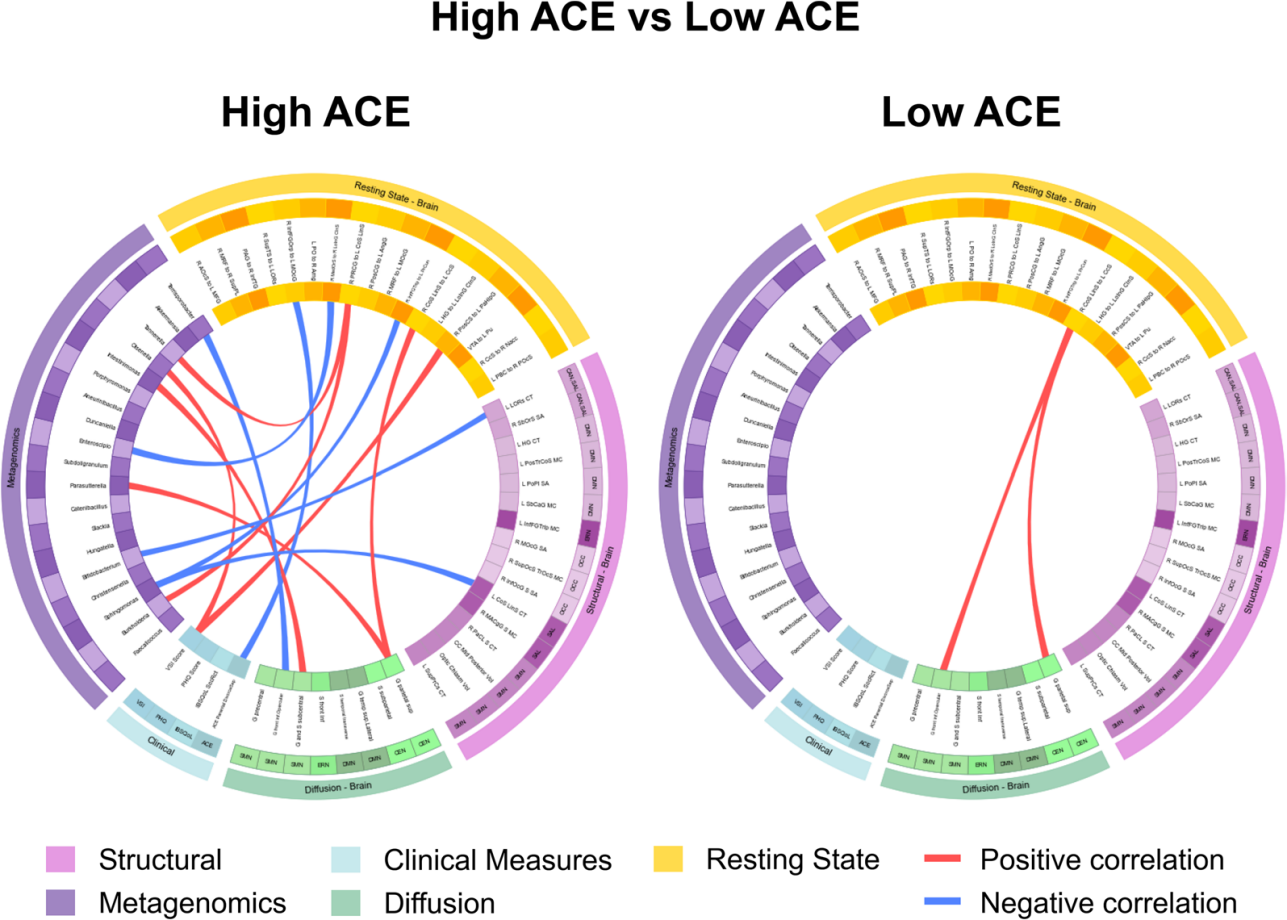
Connectograms comparing High ACE vs Low ACE HC: Healthy Control. IBS: Irritable Bowel Syndrome. N = 188 total, High ACE group n = 56, Low ACE group n = 132Dti_fa: diffusion tensor imaging Fractional Anisotropy, rspw: resting state pairwise, struc: structural, metag: metagenomic, clin: clinical ACE: Adverse Childhood Effects. VSI: Visceral Sensitivity Index. ACE Parental DivorceSep: Adverse Childhood Experiences, Parental Divorce. Brain region abbreviations for resting-state, diffusion tensor imaging, and structural scans are listed in Table 7

**Fig. 5:**
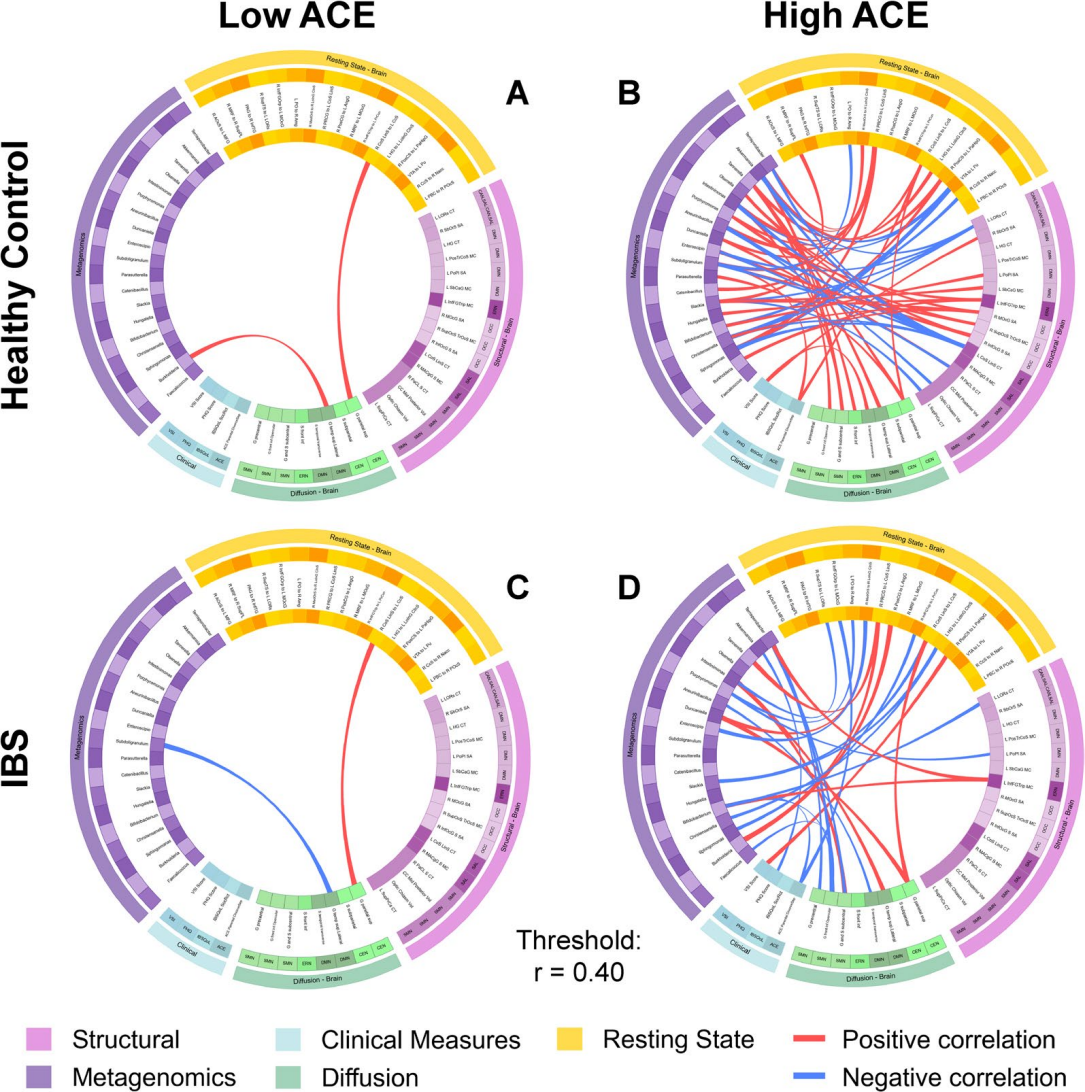
Connectograms HC: Healthy Control. IBS: Irritable Bowel Syndrome. AL: ACE Low. AH: ACE High. N = 188 total, HC low ACE group n = 77, HC high ACE group n = 119, IBS low ACE group n = 55, IBS high ACE group n = 37.Dti_fa: diffusion tensor imaging fractional anisotropy, rspw: resting state pairwise, struc: structural, metag: metagenomic, clin: clinical BH: Bowel Habits. ACE: Adverse Childhood Effects. SF12_PCS: The Physical Component Summary score of the SF-12 Health Survey. SF12_MCS: The Mental Component Summary Score of the SF-12 Health Survey. IBSQoL: Irritable Bowel Syndrome Quality of Life. VSI: Visceral Sensitivity Index. PSS Score: Perceived Stress Scale. PHQ Score: Patient Health Questionnaire Score. Brain region abbreviations for resting-state, diffusion tensor imaging, and structural scans are listed in Table 7

For each of the 4 groups, a connectogram was generated, depicting correlations between inter-omic features for the following comparisons:1 IBS vs HCs (Table 4 and Fig 3). 2. High ACE vs Low ACE (Table 5 and Fig 4), and 3. Disease and ACE related interaction effects (Table 6 and Fig 5). Associations between OMICs were found to vary between groups.

**Table 4:** Correlations between significant features by disease (IBS/HC) group

Connection	Type	Correlation	P-value
Healthy Control (N = 96)			
RS_R_CoS_LinS_to_L_CcS_TO_G_parietal_sup	dti.fa_rspw	4.16E-01	3.06E-05
IBS (N = 92)			
Bifidobacterium_TO_ACE_Parental_DivorceSep	clin_metag	−4.01E-01	7.52E-05
RS_R_CoS_LinS_to_L_CcS_TO_FA_G_parietal_sup	dti.fa_rspw	4.52E-01	1.56E-05

HC: Healthy Control. IBS: Irritable Bowel Syndrome

N = 188 total, HC group n = 96, IBS group n = 92

Dti_fa: diffusion tensor imaging Fractional Anisotropy, rspw: resting state pairwise, struc: structural, metag: metagenomic, clin: clinical

ACE Parental DivorceSep: Adverse Childhood Experiences, Parental Divorce

Brain region abbreviations for resting-state, diffusion tensor imaging, and structural scans are listed in Table 7

**Table 5:** Correlations between significant features by ACE (high/Low) group

Connection	Type	Correlation	P-value
Low ACE (n = 132)			
RS_R_CoS_LinS_to_L_CcS_TO_G_front_inf.Opercular	dti.fa_rspw	4.24E-01	6.97E-07
RS_R_CoS_LinS_to_L_CcS_TO_G_parietal_sup	dti.fa_rspw	4.21E-01	8.13E-07
High ACE (n = 56)			
Olsenella_TO_VSI_Score	clin_metag	4.23E-01	1.16E-03
RS_R_PosCS_to_L_PaHipG_TO_VSI_Score	clin_rspw	4.83E-01	3.33E-04
RS_R_InfFGOrp_to_L_MOcG_TO_ACE_Parental_DivorceSep	clin_rspw	−4.46E-01	1.05E-03
L_LORs_CT_TO_Bifidobacterium	metag_struct	−4.00E-01	2.23E-03
L_CoS_LinS_CT_TO_Sphingomonas	metag_struct	−4.59E-01	3.71E-04
G_and_S_subcentral_TO_Intestinimonas	metag_dti.fa	4.78E-01	1.94E-04
G_parietal_sup_TO_Parasutterella	metag_dti.fa	4.48E-01	5.34E-04
G_front_inf.Opercular_TO_Terrisporobacter	metag_dti.fa	−4.77E-01	2.02E-04
RS_R_PRCG_to_L_CoS_LinS_TO_Tannerella	metag_rspw	4.18E-01	2.29E-03
RS_R_MedOrS_to_R_LoInG_CInS_TO_Enteroscipio	metag_rspw	−4.67E-01	5.45E-04
RS_R_InfFGTrip_to_L_PrCun_TO_Sphingomonas	metag_rspw	−4.01E-01	3.50E-03
RS_R_PRCG_to_L_CoS_LinS_TO_Burkholderia	metag_rspw	4.01E-01	3.54E-03
RS_R_CoS_LinS_to_L_CcS_TO_G_parietal_sup	dti.fa_rspw	4.39E-01	9.11E-10

N = 188 total, High ACE group n = 56, Low ACE group n = 132

Dti_fa: diffusion tensor imaging Fractional Anisotropy, rspw: resting state pairwise, struc: structural, metag: metagenomic, clin: clinical

ACE: Adverse Childhood Effects. VSI: Visceral Sensitivity Index

ACE Parental DivorceSep: Adverse Childhood Experiences, Parental Divorce

Brain region abbreviations for resting-state, diffusion tensor imaging, and structural scans are listed in Table 7

**Table 6:** Correlations between significant features by disease (IBS/HC) and ACE (high/Low) group

Connection	Type	Correlation	P-value
Healthy Control, Low ACE			
Intestinimonas_TO_G_temp_sup.Lateral	metag_dti	3.22E-01	4.33E-03
Olsenella_TO_G_temp_sup.Lateral	metag_dti	3.36E-01	2.81E-03
Parasutterella_TO_G_front_inf.Opercular	metag_dti	3.88E-01	4.84E-04
Sphingomonas_TO_G_temp_sup.Lateral	metag_dti	3.78E-01	6.94E-04
Burkholderia_TO_G_temp_sup.Lateral	metag_dti	4.13E-01	1.87E-04
Catenibacillus_TO_RS_R_PosCG_to_L_AngG	metag_rspw	3.05E-01	7.42E-03
Christensenella_TO_RS_R_PosCS_to_L_PaHipG	metag_rspw	3.04E-01	7.52E-03
Parasutterella_TO_R_SupOcS_TrOcS_MC	metag_struct	−3.44E-01	2.20E-03
Parasutterella_TO_R_MACgG_S_MC	metag_struct	−3.38E-01	2.65E-03
Aneurinibacillus_TO_L_LORs_CT	metag_struct	−3.08E-01	6.42E-03
Sphingomonas_TO_R_SbOrS_SA	metag_struct	−3.34E-01	2.99E-03
R_SupOcS_TrOcS_MC_TO_G_temp_sup.Lateral	struct_dti.fa	−3.21E-01	7.11E-06
R_MACgG_S_MC_TO_G_front_inf.Opercular	struct_dti.fa	−3.34E-01	2.73E-06
G_front_inf.Opercular_TO_RS_R_CoS_LinS_to_L_CcS	dti.fa_rspw	3.55E-01	1.15E-06
G_temp_sup.Lateral_TO_RS_R_CoS_LinS_to_L_CcS	dti.fa_rspw	3.44E-01	2.67E-06
G_precentral_TO_RS_R_CoS_LinS_to_L_CcS	dti.fa_rspw	3.16E-01	1.68E-05
G_and_S_subcentral_TO_RS_R_CoS_LinS_to_L_CcS	dti.fa_rspw	3.33E-01	5.52E-06
G_parietal_sup_TO_RS_R_CoS_LinS_to_L_CcS	dti.fa_rspw	4.39E-01	9.11E-10
Healthy Control, High ACE			
VSI_Score_TO_S_temporal_transverse	clin_dti	5.66E-01	1.15E-02
ACE_Parental_DivorceSep_TO_Subdoligranulum	clin_metag	4.99E-01	2.95E-02
ACE_Parental_DivorceSep_TO_RS_R_CoS_LinS_to_L_CcS	clin_rspw	4.88E-01	3.97E-02
VSI_Score_TO_R_SbOrS_SA	clin_struct	4.79E-01	3.79E-02
Intestinimonas_TO_G_and_S_subcentral	metag_dti	4.76E-01	3.93E-02
Parasutterella_TO_G_parietal_sup	metag_dti	5.18E-01	2.33E-02
Tannerella_TO_G_front_inf.Opercular	metag_dti	5.20E-01	2.24E-02
Tannerella_TO_G_temp_sup.Lateral	metag_dti	6.22E-01	4.43E-03
Tannerella_TO_G_and_S_subcentral	metag_dti	5.85E-01	8.51E-03
Tannerella_TO_G_parietal_sup	metag_dti	5.89E-01	8.02E-03
Akkermansia_TO_S_front_inf	metag_dti	5.40E-01	1.70E-02
Akkermansia_TO_G_and_S_subcentral	metag_dti	5.22E-01	2.18E-02
Subdoligranulum_TO_RS_R_PRCG_to_L_CoS_LinS	metag_rspw	6.83E-01	1.77E-03
Subdoligranulum_TO_RS_L_PO_to_R_Amg	metag_rspw	−4.93E-01	3.74E-02
Intestinimonas_TO_RS_VTA_to_L_Pu	metag_rspw	−4.79E-01	4.45E-02
Intestinimonas_TO_RS_R_PRCG_to_L_CoS_LinS	metag_rspw	6.31E-01	4.98E-03
Tannerella_TO_RS_L_HG_to_L_LoInG_CInS	metag_rspw	6.01E-01	8.34E-03
Bifidobacterium_TO_RS_R_CcS_to_R_Nacc	metag_rspw	−5.41E-01	2.04E-02
Duncaniella_TO_RS_R_CoS_LinS_to_L_CcS	metag_rspw	4.86E-01	4.09E-02
Aneurinibacillus_TO_RS_R_PRCG_to_L_CoS_LinS	metag_rspw	7.36E-01	4.99E-04
Porphyromonas_TO_RS_R_PRCG_to_L_CoS_LinS	metag_rspw	4.79E-01	4.43E-02
Terrisporobacter_TO_RS_R_PRCG_to_L_CoS_LinS	metag_rspw	5.18E-01	2.76E-02
Akkermansia_TO_RS_VTA_to_L_Pu	metag_rspw	−7.88E-01	1.04E-04
Akkermansia_TO_RS_R_PRCG_to_L_CoS_LinS	metag_rspw	5.27E-01	2.47E-02
Akkermansia_TO_RS_R_PosCS_to_L_PaHipG	metag_rspw	6.36E-01	4.54E-03
Sphingomonas_TO_RS_VTA_to_L_Pu	metag_rspw	−6.33E-01	4.80E-03
Sphingomonas_TO_RS_R_PRCG_to_L_CoS_LinS	metag_rspw	7.94E-01	8.42E-05
Sphingomonas_TO_RS_R_PosCS_to_L_PaHipG	metag_rspw	5.56E-01	1.65E-02
Slackia_TO_RS_R_MRF_to_R_SupPL	metag_rspw	6.06E-01	7.67E-03
Catenibacillus_TO_RS_R_MedOrS_to_R_LoInG_CInS	metag_rspw	5.55E-01	1.68E-02
Christensenella_TO_RS_VTA_to_L_Pu	metag_rspw	−7.42E-01	4.22E-04
Subdoligranulum_TO_R_SupOcS_TrOcS_MC	metag_struct	4.78E-01	3.82E-02
Subdoligranulum_TO_L_CoS_LinS_CT	metag_struct	−5.69E-01	1.10E-02
Intestinimonas_TO_L_CoS_LinS_CT	metag_struct	−6.65E-01	1.90E-03
Olsenella_TO_R_InfOcG_S_SA	metag_struct	5.21E-01	2.23E-02
Olsenella_TO_R_MOcG_SA	metag_struct	4.90E-01	3.30E-02
Hungatella_TO_L_InfFGTrip_MC	metag_struct	5.25E-01	2.09E-02
Bifidobacterium_TO_L_PoPl_SA	metag_struct	5.34E-01	1.84E-02
Duncaniella_TO_R_PaCL_S_CT	metag_struct	5.03E-01	2.81E-02
Aneurinibacillus_TO_Optic_Chiasm_Vol	metag_struct	−4.80E-01	3.78E-02
Aneurinibacillus_TO_L_CoS_LinS_CT	metag_struct	−6.70E-01	1.71E-03
Enteroscipio_TO_R_InfOcG_S_SA	metag_struct	6.28E-01	3.97E-03
Enteroscipio_TO_L_LORs_CT	metag_struct	−4.65E-01	4.50E-02
Enteroscipio_TO_R_PaCL_S_CT	metag_struct	−4.67E-01	4.36E-02
Porphyromonas_TO_L_InfFGTrip_MC	metag_struct	5.22E-01	2.18E-02
Porphyromonas_TO_R_SupOcS_TrOcS_MC	metag_struct	4.86E-01	3.49E-02
Terrisporobacter_TO_L_CoS_LinS_CT	metag_struct	−5.07E-01	2.66E-02
Akkermansia_TO_L_LORs_CT	metag_struct	−4.91E-01	3.28E-02
Akkermansia_TO_L_CoS_LinS_CT	metag_struct	−6.41E-01	3.08E-03
Sphingomonas_TO_L_CoS_LinS_CT	metag_struct	−6.96E-01	9.29E-04
Burkholderia_TO_L_InfFGTrip_MC	metag_struct	5.43E-01	1.63E-02
Burkholderia_TO_R_MOcG_SA	metag_struct	5.60E-01	1.27E-02
Slackia_TO_L_SupPrCs_CT	metag_struct	−4.92E-01	3.22E-02
Slackia_TO_L_LORs_CT	metag_struct	−4.82E-01	3.68E-02
Slackia_TO_L_SbCaG_MC	metag_struct	5.62E-01	1.23E-02
Slackia_TO_R_MOcG_SA	metag_struct	5.17E-01	2.34E-02
Christensenella_TO_L_LORs_CT	metag_struct	−4.57E-01	4.91E-02
Christensenella_TO_L_CoS_LinS_CT	metag_struct	−5.21E-01	2.22E-02
Christensenella_TO_R_MOcG_SA	metag_struct	4.70E-01	4.21E-02
R_SupOcS_TrOcS_MC_TO_G_temp_sup.Lateral	struct_dti.fa	−3.21E-01	7.11E-06
R_MACgG_S_MC_TO_G_front_inf.Opercular	struct_dti.fa	−3.34E-01	2.73E-06
G_front_inf.Opercular_TO_RS_R_CoS_LinS_to_L_CcS	dti.fa_rspw	3.55E-01	1.15E-06
G_temp_sup.Lateral_TO_RS_R_CoS_LinS_to_L_CcS	dti.fa_rspw	3.44E-01	2.67E-06
G_precentral_TO_RS_R_CoS_LinS_to_L_CcS	dti.fa_rspw	3.16E-01	1.68E-05
G_and_S_subcentral_TO_RS_R_CoS_LinS_to_L_CcS	dti.fa_rspw	3.33E-01	5.52E-06
G_parietal_sup_TO_RS_R_CoS_LinS_to_L_CcS	dti.fa_rspw	4.39E-01	9.11E-10
IBS, Low ACE			
VSI_Score_TO_S_subparietal	clin_dti	−3.20E-01	1.74E-02
ACE_Parental_DivorceSep_TO_G_front_inf.Opercular	clin_dti	3.39E-01	1.13E-02
ACE_Parental_DivorceSep_TO_S_front_inf	clin_dti	3.43E-01	1.04E-02
ACE_Parental_DivorceSep_TO_G_precentral	clin_dti	3.54E-01	8.08E-03
ACE_Parental_DivorceSep_TO_G_and_S_subcentral	clin_dti	3.89E-01	3.31E-03
IBSQoL_SocRct_TO_G_temp_sup.Lateral	clin_dti	3.70E-01	5.36E-03
IBSQoL_SocRct_TO_G_parietal_sup	clin_dti	3.16E-01	1.88E-02
ACE_Parental_DivorceSep_TO_Faecalicoccus	clin_metag	3.19E-01	1.76E-02
VSI_Score_TO_RS_R_PosCS_to_L_PaHipG	clin_rspw	−3.08E-01	2.79E-02
Subdoligranulum_TO_G_temp_sup.Lateral	metag_dti	−4.02E-01	2.37E-03
Subdoligranulum_TO_S_front_inf	metag_dti	−3.24E-01	1.58E-02
Intestinimonas_TO_RS_R_MRF_to_L_MOcG	metag_rspw	−3.61E-01	9.17E-03
Parasutterella_TO_RS_PAG_to_R_InfTG	metag_rspw	−3.17E-01	2.35E-02
Hungatella_TO_RS_R_MedOrS_to_R_LoInG_CInS	metag_rspw	−3.71E-01	7.28E-03
Bifidobacterium_TO_RS_L_HG_to_L_LoInG_CInS	metag_rspw	−3.72E-01	7.12E-03
Duncaniella_TO_RS_R_CoS_LinS_to_L_CcS	metag_rspw	3.45E-01	1.31E-02
Terrisporobacter_TO_RS_L_PBC_to_R_POcS	metag_rspw	−3.72E-01	7.25E-03
Slackia_TO_RS_PAG_to_R_InfTG	metag_rspw	3.05E-01	2.93E-02
Catenibacillus_TO_RS_L_PO_to_R_Amg	metag_rspw	−3.13E-01	2.55E-02
Intestinimonas_TO_Optic_Chiasm_Vol	metag_struct	3.01E-01	2.57E-02
Olsenella_TO_CC_Mid_Posterior_Vol	metag_struct	3.13E-01	2.01E-02
Parasutterella_TO_R_InfOcG_S_SA	metag_struct	3.51E-01	8.66E-03
Tannerella_TO_R_SupOcS_TrOcS_MC	metag_struct	3.85E-01	3.70E-03
Tannerella_TO_L_PoPl_SA	metag_struct	3.17E-01	1.82E-02
Tannerella_TO_L_HG_CT	metag_struct	−3.66E-01	5.95E-03
Duncaniella_TO_R_MOcG_SA	metag_struct	3.19E-01	1.75E-02
Sphingomonas_TO_Optic_Chiasm_Vol	metag_struct	3.43E-01	1.05E-02
Burkholderia_TO_L_PoPl_SA	metag_struct	3.21E-01	1.69E-02
Catenibacillus_TO_Optic_Chiasm_Vol	metag_struct	3.03E-01	2.47E-02
Christensenella_TO_CC_Mid_Posterior_Vol	metag_struct	3.01E-01	2.55E-02
R_SupOcS_TrOcS_MC_TO_G_temp_sup.Lateral	struct_dti.fa	−3.21E-01	7.11E-06
R_MACgG_S_MC_TO_G_front_inf.Opercular	struct_dti.fa	−3.34E-01	2.73E-06
G_front_inf.Opercular_TO_RS_R_CoS_LinS_to_L_CcS	dti.fa_rspw	3.55E-01	1.15E-06
G_temp_sup.Lateral_TO_RS_R_CoS_LinS_to_L_CcS	dti.fa_rspw	3.44E-01	2.67E-06
G_precentral_TO_RS_R_CoS_LinS_to_L_CcS	dti.fa_rspw	3.16E-01	1.68E-05
G_and_S_subcentral_TO_RS_R_CoS_LinS_to_L_CcS	dti.fa_rspw	3.33E-01	5.52E-06
G_parietal_sup_TO_RS_R_CoS_LinS_to_L_CcS	dti.fa_rspw	4.39E-01	9.11E-10
IBS, High ACE			
VSI_Score_TO_S_temporal_transverse	clin_dti	3.58E-01	2.96E-02
ACE_Parental_DivorceSep_TO_G_front_inf.Opercular	clin_dti	3.28E-01	4.74E-02
IBSQoL_SocRct_TO_G_temp_sup.Lateral	clin_dti	3.58E-01	2.97E-02
VSI_Score_TO_Catenibacillus	clin_metag	−3.45E-01	3.67E-02
PHQ_Score_TO_Akkermansia	clin_metag	−4.07E-01	1.24E-02
ACE_Parental_DivorceSep_TO_Bifidobacterium	clin_metag	−4.41E-01	6.24E-03
VSI_Score_TO_RS_R_PosCS_to_L_PaHipG	clin_rspw	4.18E-01	1.56E-02
PHQ_Score_TO_RS_R_PosCG_to_L_AngG	clin_rspw	3.51E-01	4.53E-02
ACE_Parental_DivorceSep_TO_RS_R_InfFGOrp_to_L_MOcG	clin_rspw	−5.21E-01	1.87E-03
ACE_Parental_DivorceSep_TO_RS_R_SupTS_to_L_LORs	clin_rspw	−3.91E-01	2.44E-02
IBSQoL_SocRct_TO_RS_VTA_to_L_Pu	clin_rspw	3.56E-01	4.21E-02
VSI_Score_TO_L_HG_CT	clin_struct	3.43E-01	3.76E-02
PHQ_Score_TO_R_InfOcG_S_SA	clin_struct	−3.51E-01	3.30E-02
ACE_Parental_DivorceSep_TO_R_SupOcS_TrOcS_MC	clin_struct	−3.54E-01	3.18E-02
ACE_Parental_DivorceSep_TO_L_HG_CT	clin_struct	3.83E-01	1.92E-02
Intestinimonas_TO_G_temp_sup.Lateral	metag_dti	4.19E-01	9.92E-03
Intestinimonas_TO_G_and_S_subcentral	metag_dti	4.86E-01	2.31E-03
Olsenella_TO_G_front_inf.Opercular	metag_dti	−3.37E-01	4.17E-02
Olsenella_TO_G_and_S_subcentral	metag_dti	−3.82E-01	1.98E-02
Parasutterella_TO_G_parietal_sup	metag_dti	3.62E-01	2.78E-02
Bifidobacterium_TO_G_front_inf.Opercular	metag_dti	−4.11E-01	1.14E-02
Duncaniella_TO_G_temp_sup.Lateral	metag_dti	3.91E-01	1.67E-02
Duncaniella_TO_G_precentral	metag_dti	3.82E-01	1.98E-02
Duncaniella_TO_G_and_S_subcentral	metag_dti	3.48E-01	3.51E-02
Duncaniella_TO_G_parietal_sup	metag_dti	4.83E-01	2.48E-03
Aneurinibacillus_TO_S_temporal_transverse	metag_dti	−4.01E-01	1.40E-02
Porphyromonas_TO_G_temp_sup.Lateral	metag_dti	3.33E-01	4.43E-02
Terrisporobacter_TO_G_front_inf.Opercular	metag_dti	−7.00E-01	1.39E-06
Terrisporobacter_TO_S_front_inf	metag_dti	−3.99E-01	1.44E-02
Terrisporobacter_TO_G_precentral	metag_dti	−4.62E-01	3.98E-03
Terrisporobacter_TO_G_and_S_subcentral	metag_dti	−4.89E-01	2.12E-03
Faecalicoccus_TO_G_front_inf.Opercular	metag_dti	−4.04E-01	1.31E-02
Faecalicoccus_TO_G_parietal_sup	metag_dti	−3.44E-01	3.72E-02
Slackia_TO_G_front_inf.Opercular	metag_dti	−3.31E-01	4.56E-02
Slackia_TO_G_parietal_sup	metag_dti	−3.73E-01	2.29E-02
Intestinimonas_TO_RS_R_SupTS_to_L_LORs	metag_rspw	−4.48E-01	8.89E-03
Olsenella_TO_RS_L_HG_to_L_LoInG_CInS	metag_rspw	−3.98E-01	2.19E-02
Tannerella_TO_RS_R_PRCG_to_L_CoS_LinS	metag_rspw	4.59E-01	7.18E-03
Bifidobacterium_TO_RS_R_InfFGOrp_to_L_MOcG	metag_rspw	3.80E-01	2.90E-02
Bifidobacterium_TO_RS_R_MedOrS_to_R_LoInG_CInS	metag_rspw	−3.59E-01	4.03E-02
Duncaniella_TO_RS_R_MedOrS_to_R_LoInG_CInS	metag_rspw	4.00E-01	2.10E-02
Aneurinibacillus_TO_RS_R_InfFGOrp_to_L_MOcG	metag_rspw	3.66E-01	3.64E-02
Enteroscipio_TO_RS_L_HG_to_L_LoInG_CInS	metag_rspw	−3.84E-01	2.75E-02
Enteroscipio_TO_RS_R_MedOrS_to_R_LoInG_CInS	metag_rspw	−5.73E-01	4.86E-04
Porphyromonas_TO_RS_R_PosCS_to_L_PaHipG	metag_rspw	3.94E-01	2.35E-02
Porphyromonas_TO_RS_L_PO_to_R_Amg	metag_rspw	−4.45E-01	9.46E-03
Sphingomonas_TO_RS_R_InfFGTrip_to_L_PrCun	metag_rspw	−4.96E-01	3.29E-03
Sphingomonas_TO_RS_R_PosCG_to_L_AngG	metag_rspw	3.54E-01	4.33E-02
Burkholderia_TO_RS_PAG_to_R_InfTG	metag_rspw	3.48E-01	4.75E-02
Burkholderia_TO_RS_R_PRCG_to_L_CoS_LinS	metag_rspw	4.03E-01	2.00E-02
Burkholderia_TO_RS_R_PosCG_to_L_AngG	metag_rspw	5.24E-01	1.75E-03
Slackia_TO_RS_L_HG_to_L_LoInG_CInS	metag_rspw	−5.31E-01	1.48E-03
Slackia_TO_RS_R_CoS_LinS_to_L_CcS	metag_rspw	−5.40E-01	1.19E-03
Christensenella_TO_RS_L_HG_to_L_LoInG_CInS	metag_rspw	−4.65E-01	6.44E-03
Christensenella_TO_RS_R_InfFGTrip_to_L_PrCun	metag_rspw	−3.77E-01	3.07E-02
Christensenella_TO_RS_R_PosCG_to_L_AngG	metag_rspw	3.53E-01	4.41E-02
Subdoligranulum_TO_L_PosTrCoS_MC	metag_struct	3.88E-01	1.75E-02
Intestinimonas_TO_L_SupPrCs_CT	metag_struct	−3.50E-01	3.39E-02
Intestinimonas_TO_L_InfFGTrip_MC	metag_struct	−3.48E-01	3.46E-02
Intestinimonas_TO_R_SupOcS_TrOcS_MC	metag_struct	−3.52E-01	3.28E-02
Parasutterella_TO_R_SbOrS_SA	metag_struct	−3.28E-01	4.75E-02
Tannerella_TO_L_InfFGTrip_MC	metag_struct	−3.64E-01	2.68E-02
Hungatella_TO_L_HG_CT	metag_struct	−3.88E-01	1.77E-02
Bifidobacterium_TO_L_InfFGTrip_MC	metag_struct	4.02E-01	1.37E-02
Bifidobacterium_TO_L_LORs_CT	metag_struct	−5.14E-01	1.14E-03
Duncaniella_TO_R_PaCL_S_CT	metag_struct	−3.42E-01	3.81E-02
Enteroscipio_TO_L_CoS_LinS_CT	metag_struct	−3.32E-01	4.45E-02
Porphyromonas_TO_L_SbCaG_MC	metag_struct	−3.52E-01	3.24E-02
Porphyromonas_TO_L_PoPl_SA	metag_struct	−4.23E-01	9.10E-03
Terrisporobacter_TO_L_InfFGTrip_MC	metag_struct	4.09E-01	1.20E-02
Terrisporobacter_TO_R_SupOcS_TrOcS_MC	metag_struct	3.78E-01	2.11E-02
Akkermansia_TO_L_SbCaG_MC	metag_struct	3.53E-01	3.23E-02
Sphingomonas_TO_R_PaCL_S_CT	metag_struct	−3.26E-01	4.88E-02
R_SupOcS_TrOcS_MC_TO_G_temp_sup.Lateral	struct_dti.fa	−3.21E-01	7.11E-06
R_MACgG_S_MC_TO_G_front_inf.Opercular	struct_dti.fa	−3.34E-01	2.73E-06
G_front_inf.Opercular_TO_RS_R_CoS_LinS_to_L_CcS	dti.fa_rspw	3.55E-01	1.15E-06
G_temp_sup.Lateral_TO_RS_R_CoS_LinS_to_L_CcS	dti.fa_rspw	3.44E-01	2.67E-06
G_precentral_TO_RS_R_CoS_LinS_to_L_CcS	dti.fa_rspw	3.16E-01	1.68E-05
G_and_S_subcentral_TO_RS_R_CoS_LinS_to_L_CcS	dti.fa_rspw	3.33E-01	5.52E-06
G_parietal_sup_TO_RS_R_CoS_LinS_to_L_CcS	dti.fa_rspw	4.39E-01	9.11E-10

HC: Healthy Control. IBS: Irritable Bowel Syndrome. AL: ACE Low. AH: ACE High

N = 188 total, HC low ACE group n = 77, HC high ACE group n = 119, IBS low ACE group n = 55, IBS high ACE group n = 37

Dti_fa: diffusion tensor imaging fractional anisotropy, rspw: resting state pairwise, struc: structural, metag: metagenomic, clin: clinical

BH: Bowel Habits. ACE: Adverse Childhood Effects. SF12_PCS: The Physical Component Summary score of the SF-12 Health Survey. SF12_MCS: The Mental Component Summary Score of the SF-12 Health Survey. IBSQoL: Irritable Bowel Syndrome Quality of Life. VSI: Visceral Sensitivity Index. PSS Score: Perceived Stress Scale. PHQ Score: Patient Health Questionnaire Score

Brain region abbreviations for resting-state, diffusion tensor imaging, and structural scans are listed in Table 7

**Table 7:** Abbreviations for resting state, diffusion and structural brain MRI regions

Abbreviation	Description
Structural Key	
CC_Mid_Posterior_Vol	Corpus Callosum
Optic_Chiasm_Vol	Optic Chiasm
L_PosTrCoS	Posterior transverse collateral sulcus
R_InfOcG_S	Inferior occipital gyrus (O3) and sulcus
L_SupPrCs	Superior part of theprecentral sulcus
L_InfFGTrip	Triangular part of the inferior frontal gyrus
R_SbOrS	Suborbital sulcus (sulcus rostrales, supraorbital sulcus)
L_LORs	Lateral orbital sulcus
R_SupOcS_TrOcS	Superior occipital sulcus andtransverse occipital sulcus
L_SbCaG	Subcallosal area, subcallosal gyrus
L_CoS_LinS	Medial occipito-temporal sulcus (collateral sulcus) and lingual sulcus
L_PoPl	Planum polare of thesuperior temporal gyrus
R_MACgG_S	Middle-anterior part of thecingulate gyrus and sulcus(aMCC)
R_PaCL_S	Paracentral lobule and sulcus
R_MOcG	Middle occipital gyrus (O2, lateral occipital gyrus)
L_HG	Anterior transverse temporal gyrus (of Heschl)
Diffusion Key	
G_front_inf_Opercular	Opercular part of the inferior frontal gyrus
G_temp_sup_Lateral	Lateral aspect of thesuperior temporal gyrus
S_front_inf	Inferior frontal sulcus
G_precentral	Precentral gyrus
S_subparietal	Subparietal sulcus
G_and_S_subcentral	Subcentral gyrus (central operculum) and sulci
S_temporal_transverse	Transverse temporal sulcus
G_parietal_sup	Superior parietal lobule (lateral part of P1)
Resting State Pairwise Key	
R_MRF_to_L_MOcG	Mesencephalic reticular formation to Middle occipital gyrus (O2, lateral occipital gyrus)
VTA_to_L_Pu	Ventral tegmental area to Putamen
PAG_to_R_InfTG	Periaqueductal gray to Inferior temporal gyrus(T3)
R_InfFGOrp_to_L_MOcG	Orbital part of the inferior frontal gyrus to Middle occipital gyrus (O2, lateral occipital gyrus)
L_HG_to_L_LoInG_CInS	Anterior transverse temporal gyrus (of Heschl) to Long insular gyrus and central sulcus of the insula
R_PRCG_to_L_CoS_LinS	Precentral gyrus to Medial occipito-temporal sulcus (collateral sulcus) and lingual sulcus
R_AOcS_to_L_MFG	Anterior occipital sulcus andpreoccipital notch (temporo-occipital incisure) to Middle frontal gyrus(F2)
R_MedOrS_to_R_LoInG_CInS	Medial orbital sulcus (olfactory sulcus) to Long insular gyrus and central sulcus of the insula
R_InfFGTrip_to_L_PrCun	Triangular part of the inferior frontal gyrus to Precuneus (medial part of P1)
R_PosCS_to_L_PaHipG	Postcentral sulcus to Parahippocampal gyrus, parahippocampal part of the medial occipito-temporal gyrus(T5)
R_MRF_to_R_SupPL	Mesencephalic reticular formation to Superior parietal lobule (lateral part of P1)
R_CoS_LinS_to_L_CcS	Medial occipito-temporal sulcus (collateral sulcus) and lingual sulcus to Calcarine sulcus
L_PBC_to_R_POcS	Parabrachial complex to Parieto-occipital sulcus(orfissure)
R_CcS_to_R_Nacc	Calcarine sulcus to (Nucleus) Accumbens area
R_PosCG_to_L_AngG	Postcentral gyrus to Angular gyrus
L_PO_to_R_Amg	Pontis oralis to Amygdala
R_SupTS_to_L_LORs	Superior temporal sulcus (parallel sulcus) to Lateral orbital sulcus

#### IBS disease-related differences (Table 4, Fig 3):

IBS participants had a more negative association between *Bifidobacterium* and ACE Parental Divorce/Separation (r= −4.01x10^−1^, p=7.52x10^−5^), compared to that in HCs.

#### Adverse childhood-related differences (Table 5, Fig 4):

Compared to individuals with low ACE scores, those with high ACE scores exhibited significantly stronger and more widespread correlations between inter-omic features, including both positive and negative associations. Key findings are detailed below.

In participants with high levels of ACE, GI-related anxiety (VSI) was positively associated with connectivity between the postcentral sulcus and parahippocampal gyrus (key regions of the SMN and ERN) (r=4.83x10^−1^, p=3.33x10^−4^). In addition, high levels of parental divorce/separation on the ACE were associated with decreased connectivity in key regions of the CEN (Orbital part of the inferior frontal gyrus to Middle occipital gyrus (r=−4.46x10^−1^, p=1.05x10^−3^). Further, *Bifidobacterium* was negatively associated with the cortical thickness of left lateral occipitotemporal sulcus part of CAN/SAL (r=−4.00x10^−1^, p=2.23x10^−3^). Finally, *Sphingomonas* was negatively associated with connectivity in key regions of the SMN/DMN (Triangular part of the inferior frontal gyrus to Precuneus, r=−4.01x10^−1^, p=3.50x10^−3^).

In participants with low levels of ACE, there were positive associations between the OCC network and the SMN (superior parietal lobule, SMN, r=4.21E-01, p=8.13E-07) and a region in Opercular part of the inferior frontal gyrus (SAL, =4.21E-01, p=6.97E-07).

#### Disease and ACE-related differences (i.e., interaction effects) (Table 6, Fig 5):

In high ACE IBS participants (Fig 5d), somatic symptom severity (PHQ-15 score) was more negatively associated with *Akkermansia* (r= −4.07x10^−1^, p=1.24x10^−2^) compared to that in the other groups. Additionally, *Bifidobacterium* was more negatively associated with both ACE Parental Divorce/Separation (r= −4.41x10^-^1, p=6.24x10^−3^) and cortical thickness of left lateral occipitotemporal sulcus (L LORs CT), a region of the central autonomic network (CAN)/SAL (r= −5.14x10^−1^, p=1.14x10^−3^).

In low ACE IBS participants (Fig 5c), connectivity of the medial occipito-temporal sulcus and lingual sulcus with the calcarine sulcus [R Cos LinS to L CcS] was more positively associated with the microstructure of the superior parietal lobule [G parietal sup], a region in the central executive network (CEN) (r= 4.39x10^−1^, p=9.11x10^−10^) compared to that in the other groups. Additionally, *Subdoligranulum* was more negatively associated with the microstructure of the lateral aspect of the superior temporal gyrus (G temp sup Lateral), a region in the default mode network (DMN) (r= −4.02x10^−1^, p=2.37x10^−3^).

In high ACE HC participants (Fig 5b), multiple cortical and subcortical regions showed more positive or negative associations compared to those in other groups. This included more negative associations between the cortical thickness of the middle anterior cingulate gyrus and sulcus (L Cos LinS CT), a region in the salience (SAL) network, and the genera *Akkermansia* (r= −6.41x10^−1^, p=3.08x10^−3^), *Subdoligranulum* (p=1.10x10^−2^, r= −5.69x10^−1^), *Intestinimonas* (r= −6.65x10^−1^, p=1.90x10^−3^)*,* and *Christensenella* (r= −5.21x10^−1^, p=2.22x10^−2^). Additionally, GI symptom-related anxiety (VSI score) was more positively associated with the microstructure of the transverse temporal sulcus (S temporal transverse), a region in the DMN (r= 5.66x10^−1^, p=1.15x10^−2^) and the surface area of right suborbital sulcus (R SbOrS SA), a region in CAN/SAL (r= 4.79x10^−1^, p=3.79x10^−2^).

In low ACE HC participants (Fig 5a), *Burkholderia* was more positively associated with the lateral aspect of the superior temporal gyrus (G temp sup Lateral), a region in the DMN (r= 4.13x10^−1^, p=1.87x10^−4^) compared to that in other groups. Additionally, connectivity of the medial occipito-temporal sulcus and lingual sulcus with the calcarine sulcus (R Cos LinS to L CcS) was more positively associated with the microstructure of the superior parietal lobule (G parietal sup), a region in the CEN (r= 4.39x10^−1^, p=9.11x10^−10^).

## Discussion

The study aimed to test the hypothesis that female IBS participants differing in their reported levels of ACE (psychosocial stressors that comprise adverse SDoH occurring in childhood) are characterized by distinct biological alterations within the BGM system that can be identified by multi-omic signatures (see Graphical Abstract**)**. Key findings include: 1. High ACE participants with IBS versus their HC counterpart showed increased depression and anxiety symptoms, GI symptom-related anxiety (VSI), perceived stress levels (PSS), somatic symptom severity (PHQ-15), and had poorer physical and mental health scores (SF-12). 2. High ACE participants with IBS had distinct connectivity patterns related to beneficial gut microbiota, notably a negative association between *Akkermansia* and PHQ-15, as well as *Bifidobacterium* and ACE parental divorce/separation and salience and central autonomic networks. 3. The ensemble model accurately distinguished IBS patients with high ACE (AUC of 0.87), demonstrating strong predictive performance with an overall model accuracy of 78%. Our findings suggest that both disease status and ACE exposure independently and interactively contribute to alterations across clinical outcomes, microbiota composition, and neural network connectivity and morphology, thereby further supporting the relevance of early life stress in modulating gut-brain axis dynamics.

### IBS is associated with worse clinical outcomes, with some outcomes influenced by ACE

The greater levels of depression, anxiety, GI symptom-related anxiety (VSI), perceived stress (PSS), somatic symptom severity (PHQ-15), and poorer physical and mental health scores (SF-12) observed in IBS participants compared to healthy controls align with the biopsychosocial model of IBS. This model suggests that central dysregulation of affective and pain circuits plays a role in symptom expression [62].

Although ACEs have been associated with greater symptom severity in IBS, the present study only found trends toward higher IBS-SSS and lower QoL in IBS participants with high ACE scores. The absence of statistical significance may indicate that ACEs primarily influence extraintestinal domains such as psychological distress and stress sensitivity. This is supported by statistically significant stronger differences in somatic symptom burden.

Further, our results suggest that the influence of ACEs may be partially mediated by heightened GI symptom-related anxiety (VSI), rather than by direct physiological alterations detectable through neuroimaging or microbiome analyses. In high ACE participants, GI symptom-related anxiety (VSI) was positively correlated with connectivity between the postcentral sulcus and parahippocampal gyrus (R PosCS to L PaHipG), a region involved in interoceptive processing and perception of internal bodily sensations. Individuals with high ACE scores may exhibit heightened interoceptive awareness, amplifying their perception and anxiety related to gastrointestinal symptoms. The parahippocampal gyrus, part of the limbic system, plays a crucial role in emotional processing and stress responses. Increased connectivity in this region may reflect an exaggerated emotional response to visceral sensations, further contributing to gastrointestinal symptom-related anxiety. Studies have demonstrated that altered resting-state functional connectivity in regions responsible for sensory processing and emotional regulation is linked to visceral hypersensitivity in IBS patients [63]. This hypersensitivity, which can be exacerbated by early life stress, may lead to increased anxiety surrounding gastrointestinal symptoms.

### High ACE in IBS is characterized by dysbiosis of gut anti-inflammatory microbes, related to clinical outcomes

ACE-induced chronic stress disrupts the brain-gut-microbiota axis, leading to long-term alterations in gut microbiota composition, reduced microbial diversity, and a shift in the balance between beneficial and potentially pathogenic bacteria [64–66]. This dysbiosis contributes to immune dysregulation through altered pro-inflammatory and anti-inflammatory cytokines, fostering the chronic low-grade inflammation characteristic of IBS [66, 67]. In our cohort, IBS participants with high ACE scores demonstrated distinct microbial signatures, notably a negative correlation of beneficial genera *Akkermansia* and *Bifidobacterium* with somatic symptom severity (PHQ-15) and ACE parental divorce/separation, respectively*. Akkermansia* is linked to gut barrier integrity, immune regulation, and pain reduction [68, 69]. Previous studies have shown that treatment with pasteurized *Akkermansia* in IBS mouse models improved anxiety-like behavior, reduced colonic hypersensitivity,and reinforced intestinal barrier function [70], suggesting its potential role in modulating the gut-brain axis and reducing inflammation as a therapeutic approach. Additionally, as *Bifidobacterium* is a well-documented genus shown to improve intestinal permeability [71], alleviate IBS symptoms [72], reduce inflammatory cytokines (e.g., IL-6 and TNF-$\alpha$) [73], and modulate the gut microbiota to increase the abundance of beneficial bacteria and short-chain fatty acids (SCFAs) that are crucial for gut health [74], its depletion in high ACE IBS participants suggests a potential mechanism linking early life adversity to somatic symptom burden in IBS. This is consistent with prior research highlighting chronic stress from ACEs as exacerbating psychological distress and contributing to visceral hypersensitivity, a hallmark of IBS [75, 76].

### IBS and ACEs affect stress regulation and pain processing neural circuits, related to gut microbes

Early-life stress has been shown to contribute to central nervous system dysregulation through maladaptive neuroendocrine responses to stress, which manifest as gastrointestinal symptoms [77]. Additionally, IBS patients with a history of childhood trauma exhibit increased visceral hypersensitivity and altered brain responses to pain [78]. Our neuroimaging findings revealed significant associations between ACEs, IBS, and alterations in key neural circuits, including the salience network (SAL), sensorimotor network (SMN), default mode network (DMN), central autonomic network (CAN), and central executive network (CEN), which all play crucial roles in stress and emotional regulation and pain processing. In high ACE IBS participants, *Bifidobacterium* was negatively linked to CAN/SAL. Hyperconnectivity in the salience network is linked to hypervigilance and hyperalgesia in IBS [63] while CAN regulates stress responses by integrating physiological and emotional signals across brain regions to modulate autonomic, neuroendocrine, and behavioral adaptations [79]. Lower levels of *Bifidobacterium* may exacerbate these network dysfunctions, contributing to more severe IBS symptoms. In contrast, in low ACE IBS participants, there was a positive association between medial occipito-temporal (collateral) sulcus and lingual sulcus to calcarine sulcus and the CEN, which is involved in high-level cognitive functions [80]. This may suggest a potential compensatory mechanism within cognitive control regions to help manage IBS symptoms. Additionally, *Subdoligranulum*, a butyrate-producing beneficial bacterium, was negatively linked to the DMN, highlighting a possible role of microbiome-brain interactions in modulating pain and emotional processing in low ACE IBS participants.

In high ACE HC participants, salience (SAL) connectivity was negatively associated with beneficial gut microbial genera, including *Akkermansia, Subdoligranulum, Intestinimonas, and Christensenella*, suggesting a potential link between gut microbial composition and stress-related brain network activity. The salience network is crucial for detecting and integrating salient stimuli, including pain and emotional stress. In IBS patients, particularly those with a history of ACE, increased SAL connectivity likely contributes to heightened pain perception, emotional dysregulation, and hypervigilance [81, 82]. Additionally, *Intestinimonas,* a butyrate producing bacteria, was positively associated with the subcentral sulcus and sulci (G and S subcentral) part of the sensorimotor network connectivity. As the subcentral area encompasses parts of the primary motor and somatosensory cortices, these connectivity changes may relate to visceral sensorimotor integration and interoception, which may contribute to a latent risk for IBS. Individuals with a history of ACEs are twice as likely to have IBS than those without an ACE [6].

In summary, IBS and ACE can influence key neural circuits part of a complex interplay between early-life adversity and gut microbiota in shaping IBS pathophysiology, emphasizing the need for a more integrative approach to understanding and managing this disorder. Despite the lack of significant differences in symptom severity between IBS participants with high and low ACEs, we found unique microbiome and brain signatures. These signatures may be relevant to personalized medicine/treatment response differences. Additionally, it is possible that resilience or other protective factors buffer the effect of ACEs. However, it remains unclear whether these signatures arise from IBS itself or reflect increased vulnerability in individuals with ACEs. Future research with larger samples and a longitudinal design, following individuals with ACEs to see how ACEs can increase the vulnerability to develop IBS symptoms, is needed. Overall, these findings emphasize the critical influence of early-life stress on gut microbiota composition and support the potential of microbiome-targeted interventions in alleviating and preventing IBS symptoms in individuals with a history of ACE.

### Multi-omic integration enhances predictive modeling based on IBS diagnosis and ACEs

The ensemble model outperformed the individual base models, indicating that integrating multiple biological modalities enhances predictive power. Among the base models, the clinical model had the highest independent predictive ability (kappa = 0.67), suggesting that questionnaire-based assessments of IBS severity and symptom impact remain critical for classification. In contrast, individual neuroimaging and metagenomic models had relatively low kappa values (ranging from 0.07 to 0.18), indicating that while these features contribute to prediction, their standalone predictive power is limited. This aligns with prior work suggesting that neurobiological and gut microbial alterations in IBS are subtle and context-dependent rather than deterministic. Labus et al. (2017) indicated that while gut microbial composition differences correlate with regional brain volumes in IBS patients, these alterations are subtle and vary among individuals. Notably, AUC values varied across groups, with the highest classification performance for HCs with low ACEs (AUC = 0.98), followed by IBS patients with high ACE (AUC =0.87) and IBS patients with low ACE (AUC = 0.82). The HC high ACE group was the most difficult to classify (AUC = 0.70). The exceptionally high AUC for HC low ACE suggests that healthy individuals with minimal early-life stress exhibit the most distinct biological signatures, making them the easiest to differentiate. In contrast, the lower AUC for HC high ACE indicates that early-life adversity in healthy individuals may lead to more variable biological profiles, with heterogenous coping mechanisms, resilience factors, or subclinical symptomatology blurring distinctions from IBS groups.

### Limitations

Although this was a large, well-characterized cohort with rigorous classification into four distinct subgroups, certain limitations should be acknowledged. The sample size distribution across subgroups was uneven, with a particularly smaller representation of high ACE participants, which may impact the generalizability of findings. Additionally, the cross-sectional design precludes causal inferences regarding the relationship between ACE and multi-omic alterations. Further validation in larger, independent cohorts is necessary, including functional validation of key biomarkers. Moreover, the study was limited to female participants, restricting the ability to evaluate sex-specific differences in multi-omic profiles. The reliance on self-reported menstrual history to determine follicular phase status may be subject to recall bias and imprecise phase classification, potentially affecting the interpretation of hormonally sensitive outcomes. Further, it is possible that having a condition such as IBS in adulthood may bias the recall of ACEs. Finally, future research should compare IBS with other chronic gastrointestinal or pain conditions to further delineate biological alterations related to ACEs in IBS.

### Clinical implications and conclusions

This study highlights the magnitude of chronic stress from ACEs, which are adverse SDoH, on the multifaceted nature of IBS including psychological, neural, and gut microbiome factors that contribute to gastrointestinal symptoms in women. The study’s multi-omics approach builds on emerging research demonstrating the value of integrating clinical, neuroimaging, and microbiome data to capture the heterogeneity of IBS phenotypes. Gut microbiome and brain imaging markers can potentially be used as predictive tools for therapeutic outcomes. It can also be the key to identify therapeutic targets tailored to modulate specific microbial profiles or brain circuits to address the long-term consequences of early-life stress in individuals with IBS. Further research should comprise longitudinal studies to track dynamic changes in multi-omic markers over time, mechanistic studies to investigate causal pathways linking ACEs to brain and gut alterations, and most importantly, interventional studies to assess impact of therapies targeting microbiomes or brain connectivity on IBS outcomes. Since it is known that there are significant sex-specific differences in clinical presentation, microbiome composition, and brain-gut interactions, it would be valuable to do similar multi-omic studies with both male and females to explore these differences further.

## Data Availability

Because the data presented is part of several ongoing projects, availability of data will be made available by request.
